# Myricanol Inhibits the Type III Secretion System of *Salmonella enterica* Serovar Typhimurium by Interfering With the DNA-Binding Activity of HilD

**DOI:** 10.3389/fmicb.2020.571217

**Published:** 2020-09-25

**Authors:** Yang Wu, Xuefei Yang, Dongdong Zhang, Chunhua Lu

**Affiliations:** ^1^Key Laboratory of Chemical Biology, Ministry of Education, School of Pharmaceutical Sciences, Cheeloo College of Medicine, Shandong University, Jinan, China; ^2^Key Laboratory of Economic Plants and Biotechnology, Kunming Institute of Botany, Chinese Academy of Sciences, Kunming, China

**Keywords:** myricanol, *Salmonella*, T3SS, HilD, anti-virulence

## Abstract

The type III secretion system (T3SS) consists of a syringe-like export machine injecting effectors from the bacterial cytosol directly into host cells to establish infection. This mechanism is widely distributed in gram-negative bacteria and can be targeted as an innovative strategy for the developing of anti-virulence drugs. In this study, we present an effective T3SS inhibitor, myricanol, inspired by the use of folk medicinal plants traditionally used against infections. Myricanol is a cyclic diarylheptanoid isolated from the medicinal plant *Myrica nagi*, which is found in South and East Asia. Bioassay-guided fractionation revealed that myricanol inhibited not only the secretion of type III effector proteins of *Salmonella enterica* serovar Typhimurium UK-1 χ8956 (*S*. Typhimurium) but also the invasion of *S*. Typhimurium into mammalian cells, but showed no toxicity to bacterial growth or the host cells. RNA-Seq data analysis showed that the transcription of the pathogenesis-related SPI-1 gene was significantly inhibited by myricanol. Further study demonstrated that myricanol binds physically to HilD and interferes with its DNA-binding activity to the promoters of the *hilA* and *invF* genes. In conclusion, we propose that myricanol is responsible for the anti-infectious properties of *M. nagi* and is a novel T3SS inhibitor of *S*. Typhimurium through a previously unappreciated mechanism of action.

## Introduction

Antibiotic-resistant bacteria are an emerging threat to global public health and are threatening our ability to treat common infectious diseases. As the efficacy of antibiotics declines, the treatment of infections (such as pneumonia, tuberculosis, sepsis, gonorrhea, and foodborne illnesses) is becoming increasingly more challenging (Theuretzbacher et al., 2020). There is an urgent need for new anti-infective therapies, used alone or in combination with traditional antibiotics to prevent or treat bacterial pathogens. The Type III secretion system (T3SS) transports effector proteins into eukaryotic host cells to induce infection, which is essential for the virulence of some bacterial pathogens (Galán and Wolf-Watz, 2006; Coburn et al., 2007; Nans et al., 2015; Deng et al., 2017). The T3SS-targeted therapeutic strategies disarm pathogenic bacteria but do not directly kill them directly, thereby reducing the risk of inducing resistance. Therefore, T3SS appears to be a highly attractive target for the development of innovative agents against bacterial infections (Rasko and Sperandio, 2010).

Salmonellosis is the most frequent foodborne disease in humans. Typhoid fever caused by *S.* Typhimurium infection remains a significant health problem, especially in developing countries (Janssen van Doorn et al., 2003; Wain et al., 2015). *Salmonella enterica* serovar Typhimurium induces inflammatory diarrhea and invades non-phagocytic epithelial cells using the T3SS encoded on *Salmonella* pathogenicity island 1 (SPI-1) (Galan and Curtiss, 1989; Wallis and Galyov, 2000). The T3SS apparatus is a needle-like structure that injects bacterial effector proteins into the host cell cytosol. Structural genes for the assembly of the functional T3SS apparatus and several effector proteins are encoded in the SPI-1 *prg/org*, *inv/spa*, and *sic/sip* operons, and several transcriptional regulators are also encoded by SPI-1. Three AraC-like regulators, HilD, HilC, and RtsA, control expression of *hilA*, and thereby induction of the SPI-1 system (Lostroh and Lee, 2001; Ellermeier et al., 2005). HilD is the dominant regulator of the system, while HilC and RtsA work as amplifiers of the signal (Saini et al., 2010; Petrone et al., 2014; Sang et al., 2017; Narm et al., 2020). There is a diagrammatic scheme shown in Figure 4D. Furthermore, HilD is also known to function in the expression of SPI-2 (Bustamante et al., 2008). Intriguingly, loss of HilD function substantially decreased bacterial virulence against the host (Sang et al., 2017). These findings led to the hypothesis that targeting HilD may impair the virulence of *S.* Typhimurium.

Medicinal plants, several of which traditionally used in anti-infectious, are a treasure trove for drug discovery. Some flavonoids, such as baicalein and quercetin, from traditional Chinese medicines, inhibit the invasion of *S.* Typhimurium into epithelial cells by blocking T3SS effectors and translocases (Tsou et al., 2016). TS027 and TS103, identified from a library of plant phenolic compounds, inhibit the transcription of *exoS* virulence regulator in *Pseudomonas aeruginosa* (Yamazaki et al., 2012). Thymol, a monoterpene phenol derivative of cymene, inhibits the translocation of SipA into HeLa cells by *S.* Typhimurium (Zhang et al., 2018). Resveratrol tetramer (-)-hopeaphenol was identified as an effective inhibitor of the T3SS in *Yersinia pseudotuberculosis* and *Pseudomonas aeruginosa* (Zetterstrom et al., 2013). However, although the molecules that have been discovered so far are inspiring, our knowledge of their potential use as medicines remains nevertheless preliminary, paving the way for future research in this area. In most cases, these molecules were identified by a simple screening based on the inhibition of gene transcription, and the molecular targets of the inhibitors remain to be identified.

*Myrica nagi* is a traditional medicinal plant with anti-diarrheal and gut modulatory activities broadly used in Myanmar (Aleem et al., 2015). Our previous screening results suggested that five of 93 medicinal plant ethanol extracts displayed inhibitory activities against the secretion of *Salmonella* T3SS effector proteins (Li et al., 2018). *Myrica nagi* is the one with inhibitory activity. To identify the molecules with anti-T3SS activity from the bark extract of *M. nagi*, in this study, the anti-T3SS guided isolation was carried out which led to the identification of myricanol (**1**) and monomethyl myricanol (**3**) with inhibitory activity against T3SS effector secretion. We also explored the potential mechanism of myricanol action against T3SS. Together, these results suggested that myricanol could serve as a candidate for the development of T3SS-targeted therapeutic agents.

## Materials and Methods

### Bacterial Strains, Plasmids, and Growth Conditions

The bacterial strains and plasmids used in this work are listed in Supplementary Table S1. *Salmonella enterica* serovar Typhimurium UK-1 χ8956 (ΔP*rpoS*183::TT araC PBAD *rpoS*) (Curtiss et al., 2009) was grown in Luria-Bertani (LB) broth or on LB agar plates (LB broth with 1.5% w/v agar) supplemented with 0.2% L-arabinose at 37°C or 28°C. *S*. Typhimurium carrying the vector of pBAD-*hilA*/*hilD*-pWSK29 was cultured in LB broth supplemented with 0.2% L-arabinose and ampicillin (100 μg/ml) to induce the over-expression of HilA or HilD.

### Plant Material and Isolation of Compounds 1–10 From *M. nagi* Bark Extract

*Myrica nagi* was purchased from Zay Cho Market in April 2017 after a preliminary ethnobotanical survey. It was authenticated by Yu Zhang, and a voucher specimen (EB2017KLW051) was deposited in Kunming Institute of Botany, Chinese Academy of Sciences.

Dried *M. nagi* barks (500 g) were macerated 2 days with 3 L of ethyl acetate (EA). After maceration for three times, the EA extract was partitioned between MeOH and petroleum ether (PE) to obtain PE and MeOH extracts. Then, MeOH extract was subjected to Sephadex LH-20 to yield fractions S1–S6. Fraction S1 and S2 (89 mg) were further purified by silica gel column chromatography (CC) eluted with CH_2_Cl_2_/MeOH to provide **8** (21.3 mg). Fraction S3 was fractionated by medium pressure liquid chromatography eluted with H_2_O and 30, 50, 70, and 100% MeOH (2 L each) to obtain fractions S3.F1–F7. Fractions S3.F1–F2 from 30% MeOH were combined and purified by silica gel CC eluted with CH_2_Cl_2_/MeOH to yield **4** (12.3 mg) and **5** (22.9 mg). The fractions S3.F3–F4 from 50% MeOH were combined and were further purified by silica gel CC (CH_2_Cl_2_/MeOH) to obtain **6** (43.2 mg) and **7** (120.2 mg). Fraction S3.F5 from 70% MeOH were further purified by CC (PE/EA) to provide **1** (1.3 g), **2** (64.1 mg) and **3** (20.3 mg). Fraction S3.F7 was subjected to preparative HPLC eluted with 45% CH_3_CN to afford **9** (*t*_*R*_ 15 min, 12.5 mg) and **10** (*t*_*R*_ 9.2 min, 9.1 mg). Compounds **1** and **3** were obtained by anti-T3SS guided fractionation. Their structures were identified based on their ^1^H, ^13^C NMR (see in the Supplementary Material), ^1^H-^1^H COSY, HMQC, and HMBC spectroscopic data and compared with the reported data. Cytosporone B (Csn-B) was chosen as the positive-control compound used in this study (Li et al., 2013). All compounds were dissolved in DMSO.

### Isolation and Detection of Secreted Proteins

SPI-1-associated effector proteins were isolated and detected as described by Hudson et al. (2007) and Negrea et al. (2007). The assay relies on the fact that when *Salmonella* grows under T3SS-inducing conditions (a temperature shift from 28 to 37°C, which simulates the temperature change for *Salmonella* when infecting human hosts), it secretes effector proteins (Sips and Sops) via T3SS into the culture supernatant. The overnight cultures at 28°C of *S.* Typhimurium were diluted 10-fold in fresh LB (0.2% L-arabinose) for 4 h at 37°C/220 rpm in the presence of the compounds at the indicated concentrations. Secreted proteins in 1 mL supernatant were precipitated for 40 min with a final concentration of 10% TCA at 4°C. Secreted effectors were collected at 14000 × g for 20 min. Then, they were washed twice with 300 μL of ice-chilled acetone, and the precipitates were allowed to dry for 15 min before an appropriate volume of 4% SDS loading buffer was added. The samples were immediately heated for 10 min at 95°C to denature the proteins and were subsequently separated using 10% SDS-PAGE and stained with Coomassie blue.

### Measurement of Bacterial Growth

*S.* Typhimurium cells grown overnight in LB broth at 28°C were diluted 10 times in fresh LB (0.2% L-arabinose) and incubated for 9 h in the presence or absence of myricanol at 200 μM. The OD_600_ of the culture was measured by a microplate reader (TECAN) every hour. Three replicates were measured in each experiment.

### Cytotoxicity Assays

CCK-8 assays were performed to evaluate the effect of the compounds on the growth rate of the host cells. The SW480 cells were plated into 96-well plates at a density of 1.0 × 10^4^ cells per well and incubated in RPMI 1640 medium with 10% FBS at 37°C and 5% CO_2_ for 24 h. After washing three times with PBS, cells were treated with different concentrations of myricanol for 12 h. Then, the supernatant was discarded. After washing three times, PBS with 10% CCK-8 reagent (Hanbio) was added to each well and the plates were incubated at 37°C for 3.5 h. The cell viability was determined by measuring the OD_450_.

### Cell Culture, Bacterial Infection, and Immunofluorescence

SW480 cells were cultured in 12-well plates in RPMI 1640 (Biological Industries) supplemented with 10% FBS at 37°C in a humidified incubator with 5% CO_2_. Meanwhile, *S.* Typhimurium was cultured overnight at 28°C/220 rpm. The overnight culture of *S.* Typhimurium was diluted 50 times in LB (0.2% L-arabinose) in the presence or absence of the compounds at the indicated concentrations and cultured for 4 h at 37°C. Cells were infected with *S.* Typhimurium at a multiplicity of infection (MOI) of 50 at 37°C for 40 min. Cells were then washed three times with PBS and placed in fresh RPMI 1640 medium supplemented with 100 μg/mL gentamicin for 1 h at 37°C. For the gentamicin protection assay, after incubation with 100 μg/mL gentamicin for 1 h, the cells were washed three times with PBS and then lysed with 0.1% Triton X-100 solution. The lysates with the appropriate dilution were plated on LB agar plates to count the CFU of bacteria. For the immunofluorescence assays, cells were fixed with 4% paraformaldehyde in PBS for 10 at room temperature followed by permeabilization with ice-cold 0.5% Triton X-100 in PBS for 20 min. Cells were then blocked with 3% BSA in PBS for 1 h at room temperature. The anti-*Salmonella* LPS antibody (Abcam, 1: 500 dilution) was incubated overnight with the cells at 4°C. After washing three times in PBS, a FITC-conjugated goat anti-mouse IgG H&L (Abcam, 1: 500 dilution) was used as the secondary antibody. The observation was conducted using a Zeiss fluorescence microscope.

### RNA-Seq and Data Analysis

*S.* Typhimurium was cultured overnight at 28°C with shaking. The following day, the cultures were diluted 100-fold in LB broth (0.2% L-arabinose) with 100 μM myricanol or solvent for 4 h. Total RNA was isolated and treated with TURBO DNase (Ambion) to remove residual genomic DNA and samples were submitted to the Beijing Genomics Institute for library preparation and sequencing. The concentrations of the extracted RNA samples were determined using a NanoDrop system (NanoDrop, Madison, United States), and the integrity of the RNA was examined using the RNA integrity number (RIN) on an Agilent 2100 bioanalyzer (Agilent, Santa Clara, United States). Single-end reads, each with a read-length of 50 bp, were sequenced on a BGISEQ-500 sequencer. Clean reads were filtered using the SOAPnuke software and aligned to the *S.* Typhimurium genome (NC_016863.1), and differentially expressed genes were then analyzed using a Dr. TOM system. The transcriptome data have been submitted to NCBI with the SRA accession number PRJNA639098.

### Real-Time PCR

*S.* Typhimurium was cultured overnight at 28°C with shaking. The following day, cultures were diluted 100-fold in LB broth (0.2% L-arabinose) in the presence or absence of myricanol and grown at 37°C/220 rpm for 4 h. The total RNA was extracted using a TRIzol Plus RNA purification kit (Invitrogen). The total RNA was reverse transcribed into cDNA using the RevertAid first-strand cDNA synthesis kit (Fermentas). The PCRs were performed in 25 μL volumes using SYBR Premix Ex Taq TM (Takara). The PCR amplification was assessed using a 7000 Sequence Detection System (Applied Biosystems). All samples were analyzed in triplicate, and the primer pairs used are listed in Supplementary Table S2.

### Western Blot Analysis

The lysate of *S.* Typhimurium was heated for 10 min at 95°C to denature the proteins and separated by 10% SDS-PAGE. The proteins in SDS-PAGE gels were transferred to the right size PVDF (polyvinylidene fluoride) membrane with a wet transfer apparatus (Bio-Rad). The membranes were blocked for more than 1 h with 5% w/v BSA (bovine serum albumin) in TBST (Tris–buffered saline mixed with Tween 20) at room temperature with shaking. The membrane was incubated in 5% BSA supplemented with anti-SipC monoclonal antibody (tgcBioMICS, 1:5000 dilution), anti-FliC monoclonal antibody (Invivogen, 1: 5000 dilution), anti-HilA monoclonal antibody (CUSABIO, 1:5000 dilution), or anti-DnaK polyclonal antibody (CUSABIO, 1:2000 dilution)^1^ overnight at 4°C. The membranes were washed three times with TBST and indicated HRP-conjugated secondary antibody for 1 h at room temperature. Immunoblots were visualized by a chemiluminescence detection kit (Tanon Science & Technology). Chemiluminescent detection was performed using a ChemiDoc XRS gel imaging system (BioRad) and detected with a chemiluminescent system (BioRad) and quantified by a semi-quantification software, ImageJ.

### Protein Purification

*Escherichia coli* strain BL21 was transformed with the plasmid pET28a-*hilD* and then grown in LB medium containing 100 μg/mL ampicillin at 37°C. Then, 0.5 mM IPTG was added to induce the expression of *hilD* when the OD_600_ reached 0.8, and the cells were then incubated at 18°C for 14 h. They were then harvested and resuspended in lysis buffer (8 mM Na_2_HPO_4_, 2 mM NaH_2_PO_4_, 350 mM NaCl, 5% glycerol), and then broken using a low-temperature ultra-high pressure cell disrupter. After centrifugation, the soluble protein was loaded onto a 1 mL Ni-NTA column and incubated at 4°C for 1 h. The column was washed with lysis buffer containing 20 mM imidazole, and the bound protein was eluted with lysis buffer containing 200 mM imidazole. The eluted protein was then further purified by gel filtration (Superdex 200, GE Healthcare) and subsequently stored at −80°C.

### Electrophoretic Mobility Shift Assay (EMSA)

The electrophoretic mobility shift assay (EMSA) experiments were carried out as previously described (Hellman and Fried, 2007). The DNA-binding ability of HilD to the *hilA* and *invF* promoters was analyzed using EMSA. The promoter region of *hilA* (corresponding to −339 to +25 bp), *invF* (corresponding to −441 to +15 bp) and *lon* (a 297 bp nonspecific DNA fragment) were amplified using FAM-labeled primers (listed in Supplementary Table S2) from *S.* Typhimurium genomic DNA. A 5 pmol sample of the DNA fragment was mixed with 0.5 μg purified HilD in a binding buffer (20 mM HEPES pH 7.2, 20 mM KCl, 1% glycerol, 1 mM DTT, 0.04 mM EDTA, 0.01% Triton X-100). Myricanol was added at increasing amounts, then incubated at 25°C for 30 min. The samples were separated on a 6% DNA retardation gel using 0.5 × TBE running buffer.

### Thermal Shift Assay (TSA)

The thermal shift assay (TSA) experiments were carried out as previously described (Molina et al., 2013). The purified protein was diluted to the appropriate concentration. Myricanol and HilD were mixed at an equimolar ratio, and the mixture was incubated for 1 h at room temperature. A 50 μL volume of each sample was transferred to a wells of a PCR plate and heated for 3 min at temperatures ranging from 44°C to 62°C and then cooled for 3 min at room temperature. After cooling, the samples were centrifuged for 20 min at 12000 × *g* immediately. The soluble proteins were separated using 10% SDS-PAGE and stained with Coomassie blue.

### Drug Affinity Responsive Target Stability (DARTS) Assay

Drug affinity responsive target stability (DARTS) assays were carried out as previously described (Lomenick et al., 2009). *S.* Typhimurium with the plasmid of pBAD-*hilD*-s-pWSK29 was induced using 0.2% L-arabinose for 4 h. Cell lysates were isolated in TNC buffer (50 mM Tris–HCl, pH 8.0, 50 mM NaCl, 10 mM CaCl_2_) at the indicated concentration, followed by incubation with 50 μM myricanol or DMSO for 1 h at room temperature, and then Pronase E (Sigma-Aldrich) was added to the lysates at protein ratios of 1:25600, 1:12800 and 1:6400 for digestion for 20 min at 37°C. Reactions were stopped by adding SDS-PAGE loading buffer and were analyzed using western blotting.

### Microscale Thermophoresis (MST) Assay

The microscale thermophoresis (MST) assay was carried out as previously described (Wienken et al., 2010). HilD was first labeled with a fluorescent dye. Then, 12-point serial dilutions of myricanol (0.48 to 2 × 10^5^ nM) were prepared in PBS buffer (8 mM Na_2_HPO_4_, 2 mM NaH_2_PO_4_ pH 7.2; 340 mM NaCl, 0.05% Tween-20) supplemented with 2% DMSO and mixed with the 100 nM HilD solution. Reaction mixtures were loaded into standard treated capillaries and analyzed using MST at medium MST power and 100% LED power with a laser-on time of 5 s. The K*_*d*_* was calculated by taking the average of triplicate F_*norm*_ measurements at each concentration and fitting the data to a sigmoidal four-parameter fitting function in Prism (GraphPad Software).

## Results

### Myricanol (1) Is a T3SS Inhibitor of *S.* Typhimurium

**FIGURE 1:**
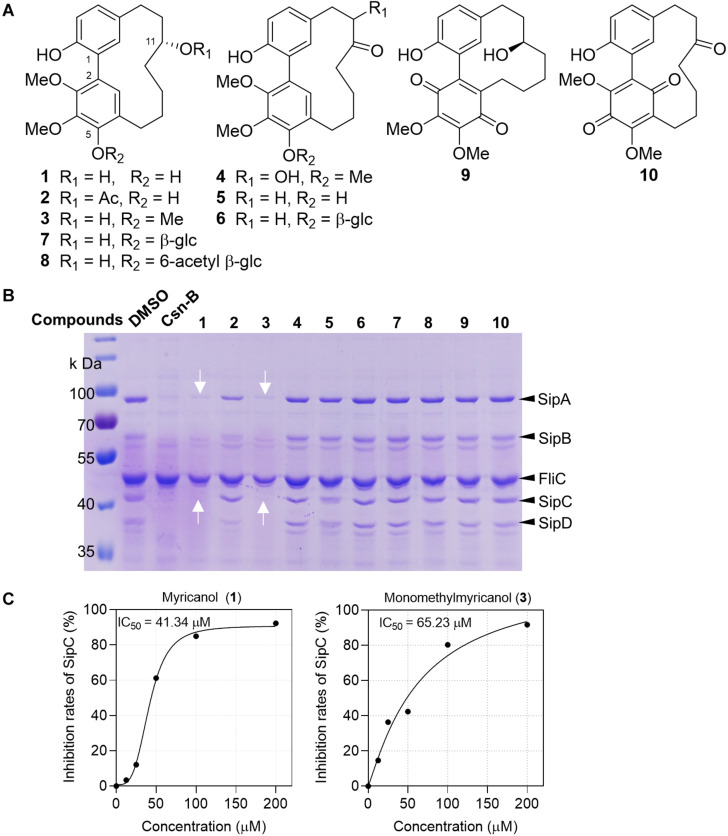
Myricanol (**1**) is an effective T3SS inhibitor of *S.* Typhimurium. **(A)** The chemical structures of **1**–**10** isolated from *Myrica nagi.* **(B)** *S.* Typhimurium was grown under T3SS-inducing conditions with 100 μM Csn-B, compounds **1–10,** or an equivalent volume of DMSO. Effector proteins of T3SS secreted into the culture supernatant were assessed by precipitating the secreted proteins and visualizing with Coomassie blue. Csn-B, positive control compound. The white arrows indicate the position of the SipA and SipC proteins. **(C)** The IC_50_ values of compounds **1** and **3**. The SipC effector protein levels in the broth were quantified by image J software and the IC_50_ was determined using the GraphPad Prism 7 software.

Inspired by the folk-medicinal use of plants as anti-infective agents, our group screened 93 crude extracts of medicinal plants from Myanmar, and five extracts could significantly suppress the secretion of SPI-1 effector proteins (Li et al., 2018). Compounds **1**–**10** (Figure 1A) purified from the bark extract of *M. nagi* were incubated with *Salmonella* for 4 h. The proteins secreted into the supernatant were precipitated using 10% trichloroacetic acid (TCA) and separated using 10% SDS-PAGE and then visualized either by staining with Coomassie blue or with western blots. The band of protein expression was quantified using Image J software and normalized to the FliC expression level. The concentrations of *Salmonella* SPI-1 effector proteins (SipA, SipB, SipC, and SipD) in the broth were significantly reduced by the treatments with myricanol (**1**) and monomethyl myricanol (**3**) at 100 μM (Figure 1B). Myricanol and monomethyl myricanol inhibited the secretion of SipC in a dose-dependent manner at a range of concentrations from 0 to 200 μM under SPI-1-induced conditions. Quantification of the SipC effector protein levels in the broth after treatment with different concentrations of myricanol and monomethyl myricanol was conducted using western blotting (Supplementary Figure S2). The 50% inhibitory concentration (IC_50_) value of **1** was calculated to be 41.34 μM, which is lower than the IC_50_ value of **3** (65.23 μM) (Figure 1C). Therefore, myricanol presented a better inhibitory activity than **3**, the 5-*O*-methylated myricanol. In addition, 5-glycosyl substituted compounds (**7** and **8**) displayed poor inhibitory activity. Thus, it seems that the free hydroxyl at C-5 is advantageous for optimal activity. Moreover, 11-acetyl substituted (**2**) and myricanone (**5**) showed significantly decreased activity compared to myricanol, which suggested that the 11-hydroxy group is essential for the T3SS inhibition. The growth curve showed that **1** and **3** did not significantly inhibit the bacterial growth at the concentration of 200 μM (Supplementary Figure S1). Those results illustrated that the specific cyclic diarylheptanoids **1** and **3** from *M. nagi* contributed to the anti-virulence effect against *S.* Typhimurium.

### Myricanol Inhibited *S.* Typhimurium Invasion Into SW480 Cells

**FIGURE 2:**
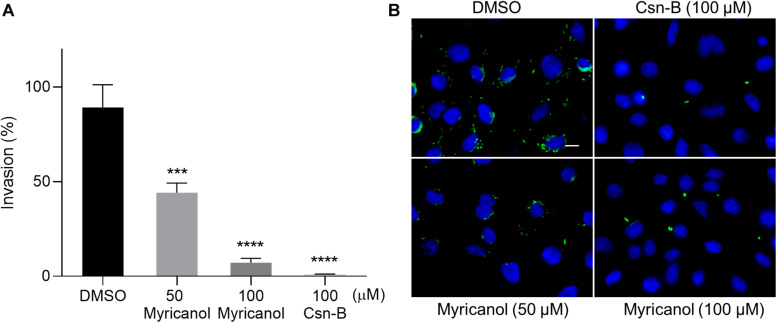
Myricanol inhibited the invasion of *S.* Typhimurium into SW480 cells. **(A)** Myricanol inhibited the invasion of *S.* Typhimurium into SW480 cells, as measured by a gentamicin protection assay. Myricanol was added to the bacterial cultures for 4 h before these were used for infection. The rates of the invasion were calculated by setting the values of the internalized bacteria in untreated samples as 100%. The indicated *p*-values were determined by one-way ANOVA. (Mean ± S.D., *n* = 3, ***P < 0.001, ****P < 0.0001). **(B)** Immunofluorescence analysis of intracellular *S.* Typhimurium in the SW480 cells. Green staining indicates *Salmonella* and blue indicates nuclei. Scale bar = 10 μm.

During infection, the SPI-1 coded injectisome and effectors are known to mainly induce the rearrangement of host cell actin cytoskeleton to form membrane ruffling, thereby allowing the entry of the bacteria into non-phagocytic cells (Du Toit, 2015; Pinaud et al., 2018). We evaluated the effects of myricanol on bacterial invasion into SW480 cells using a gentamicin protection assay. Culturing the bacteria with 100 μM myricanol for 4 h before infection reduced the rate of invasion to less than 10% (Figure 2A). Intracellular *S.* Typhimurium labeled with FITC-conjugated goat anti-mouse secondary antibody (marked in green) could also be readily observed by immunofluorescence analysis (Figure 2B). These results showed that myricanol significantly reduced bacterial entry into SW480 cells, consistent with its ability to inhibit SPI-1 effectors. Furthermore, the CCK-8 results showed that myricanol had no significant toxicity for SW480 cells at concentrations as high as 200 μM (Supplementary Figure S3).

### Myricanol Reduces the Transcription of T3SS Genes

**FIGURE 3:**
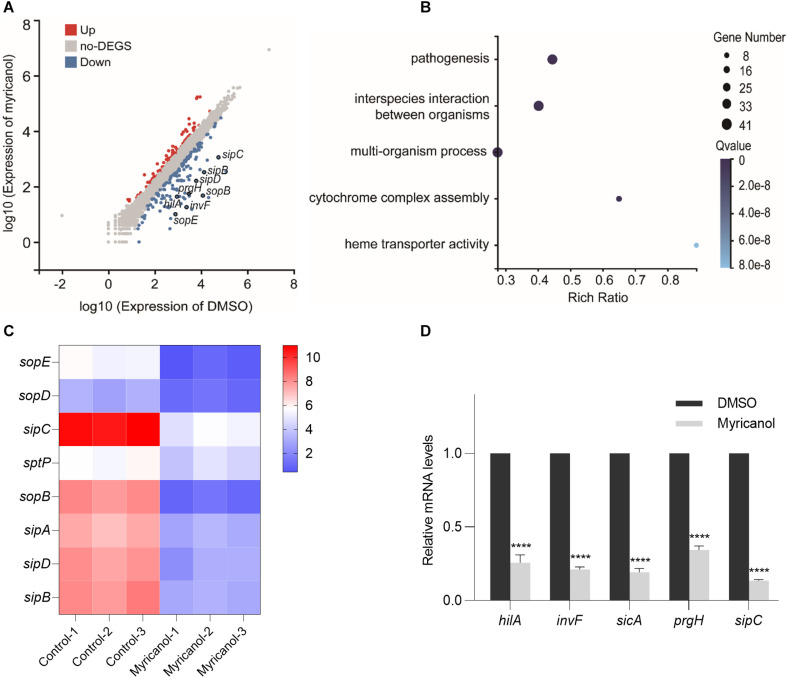
Analysis of RNA-Seq data. **(A)** Scatter plot analysis of differentially expressed genes of *S.* Typhimurium transcripts in the presence of myricanol. FDR ≤ 0.001 and Log_2_FC ≥ 1 were used as the threshold to evaluate the significance of differences in gene expression. Red and blue dots represent up- or downregulated transcripts, respectively, and gray dots represent transcripts without significant changes under myricanol treatment. **(B)** Gene Ontology (GO) enrichment analysis of differentially expressed genes in *S.* Typhimurium under the treatment of myricanol or DMSO. The Rich Ratio = Term Candidate Gene Num/Term Gene Num; the *Y*-axis is GO Term; the size of the bubble indicates the number of differential genes labeled as a particular GO Term; the color represents the enriched *Q* value. **(C)** Heat map analysis of 8 differentially expressed genes enriched in the bacterial invasion pathway of epithelial cells. The *X*-axis represents the log_2_(FPKM+1) of the sample; the *Y*-axis represents the gene. The red and blue colors indicate high and low expression, respectively. **(D)** The relative expression levels of five differentially expressed genes in myricanol- or solvent-treated were determined by real-time PCR. The indicated *p*-values were determined by the standard Student’s *t*-test. (Mean ± S.D., *n* = 3, ****P < 0.0001).

*S*. Typhimurium was cultured with or without myricanol at 100 μM for 4 h. The total RNA was then extracted and subjected to RNA-seq. With a cutoff of FDR ≤ 0.001 and fold change ≥2, 243 genes were identified as significantly differentially expressed genes. Of these, 89 genes were upregulated and 154 genes were downregulated by myricanol (Figure 3A). A large proportion of genes identified in the category “biological processes” were involved in pathogenesis, interspecies interactions between organisms, multiorganism processes, and cytochrome complex assembly (Figure 3B). Pathway enrichment analysis with KEGG was performed on these genes. Biological processing during bacterial invasion of epithelial cells was the most represented biochemical pathway, and the eight enriched genes belonging to this pathway were all downregulated by myricanol (Figure 3C). Gene expression of the pathogenesis-related SPI-1 was significantly downregulated in those pathways (e.g., *prg*/*org* and *inv*/*spa*, encoding the needle complex; *sic*/*sip*, encoding the effector proteins, and the translocon *hilACD*, encoding the transcriptional regulators) (Supplementary Table S3). To confirm the RNA-Seq results, five of the selected genes (*hilA*, *invF*, *sicA*, *sipC*, and *prgH*) involved in *Salmonella* infection were analyzed using a real-time PCR assay. The real-time PCR results showed that the relative mRNA levels of all of the tested genes decreased, which was a similar trend in differential expression to the RNA-Seq data (Figure 3D). We speculated that the reduction of the effector protein in the broth was not only due to the destruction of the syringe structure of T3SS by myricanol. This result guided us to explore the upstream regulatory factors of the T3SS apparatus and its effectors.

### Myricanol Inhibits the T3SS Mainly by Lowering the Level of HilA

**FIGURE 4:**
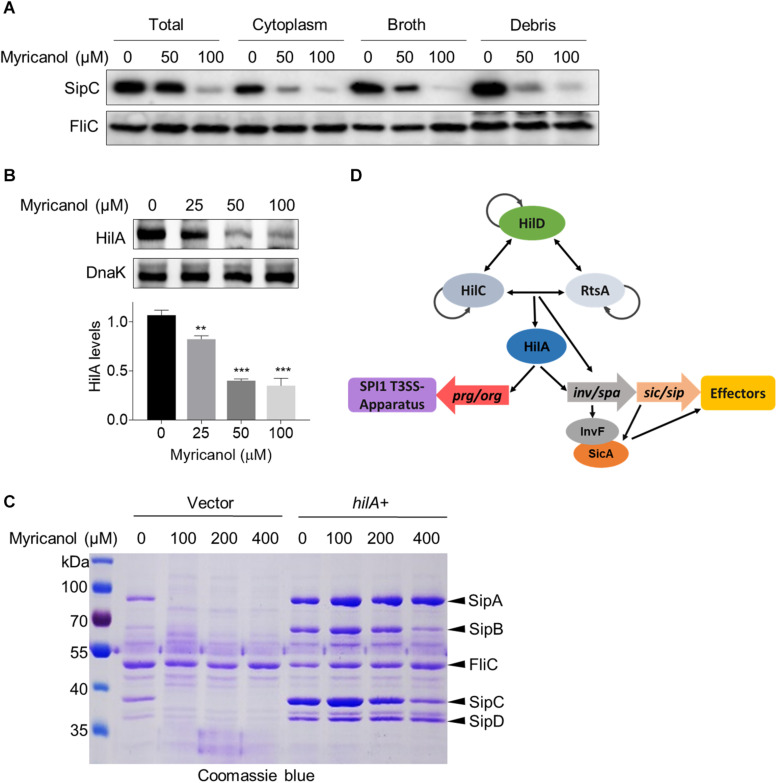
Myricanol inhibits T3SS of *S.* Typhimurium primarily by reducing the level of HilA. **(A)** *S.* Typhimurium was cultured with myricanol for 4 h. The levels of SipC in the broth, cytoplasm and cell membrane (Debris) were analyzed by western blot. SipC levels in all fractions were significantly decreased. **(B)** HilA levels were reduced by myricanol in *S.* Typhimurium. (Mean ± S.D., *n* = 3, **P < 0.01; ***P < 0.001). **(C)** Myricanol treatment decreased the protein level of SipA, SipB, SipC, and SipD in the broth, which was restored by HilA overexpression. **(D)** Working model for SPI1 regulation.

Myricanol reduced the concentrations of the secreted effectors in the culture supernatant as described above. We first investigated the levels of SipC in the culture supernatant, cytoplasm and cell membrane of *S.* Typhimurium. The western blot results showed that the SipC levels of all fractions were significantly decreased by myricanol, but were not completely blocked in the cytosol (Figure 4A). Next, we tested whether the reductions in effector concentrations were caused by protein degradation. We found that the decrease in SipC was hardly reversed by the addition of a protease inhibitor cocktail (Supplementary Figure S4), suggesting that myricanol did not regulate the SipC level in a protease-dependent manner. HilA, the critical regulator of SPI-1, induces the expression of the AraC-like regulator genes *invF* and *sicA*, encoding the *inv*/*spa*, *prg*/*org*, and *sic*/*sip* operons (Ellermeier et al., 2005; Golubeva et al., 2012). Because of this, we investigated HilA concentrations in the presence or absence of myricanol at different concentrations. Western blot analysis showed that the HilA levels in *S.* Typhimurium significantly decreased in the presence of myricanol in a dose-dependent manner (Figure 4B). Additionally, myricanol administration decreased the protein level of SipA, SipB, SipC, and SipD in the broth, which was significantly restored by HilA overexpression (Figure 4C). These results suggest that myricanol inhibits the T3SS of *S.* Typhimurium mainly by lowering the level of HilA.

### Myricanol Interferes With the DNA-Binding Ability of HilD

**FIGURE 5:**
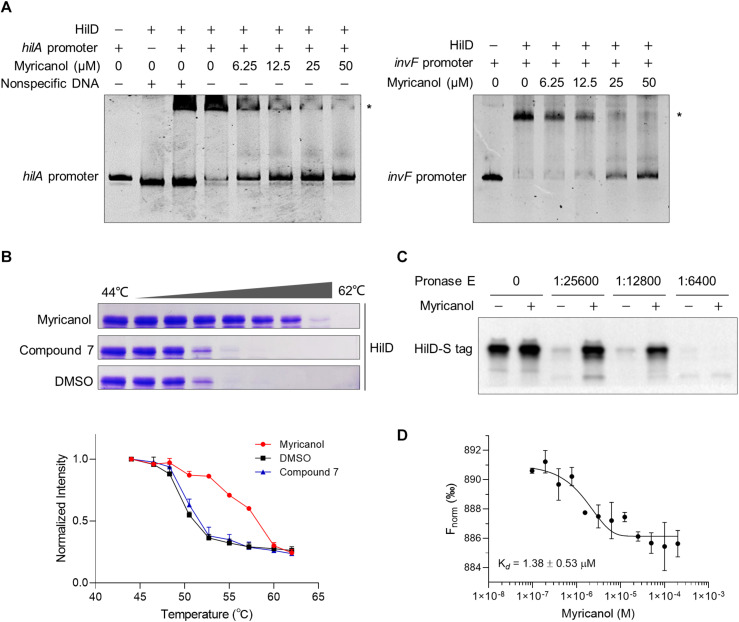
Myricanol is physically bound to HilD and interfered with the DNA-binding activity of HilD. **(A)** Myricanol inhibited the DNA-binding ability of HilD to the *hilA* and *invF* promoters as assessed using an electrophoretic mobility shift assay (EMSA). **(B)** Myricanol and HilD were mixed in an equimolar ratio, and the mixture was incubated for 1 h. The samples were incubated at temperatures ranging from 44°C to 62°C for 3 min. The soluble proteins were subjected to 10% SDS-PAGE and then Coomassie-stained. The myricanol increased the thermal stability of the HilD protein in thermal shift assays (TSA). Compound **7**, negative control. **(C)** Drug affinity responsive target stability (DARTS) confirmed that myricanol physically bound to the HilD protein. The expression of *s-hilD* was induced by 0.2% L-arabinose in *S.* Typhimurium, and the bacterial cell lysate was treated with myricanol, followed by pronase E digestion and western blot analysis. **(D)** The binding of myricanol to HilD was quantified using MST. The data yield a dissociation constant K*_*d*_* = 1.38 ± 0.53 μM.

Following induction of SPI-1, three homologous proteins, RtsA, HilC, and HilD, form a complex feed-forward regulatory loop to control SPI-1 expression (Narm et al., 2020). Of these proteins, HilD is the dominant regulator, while HilC and RtsA work as amplifiers of HilD, which is also self-regulated (Schechter and Lee, 2001). HilD is also able to bind to the promoters of *hilA* and *invF* to active SPI-1 (Schechter and Lee, 2001). In the supernatant of the *hilD*-overexpressing strain, the concentrations of SipA, SipB, SipC, and SipD were all increased compared with the control. Moreover, the sensitivity of the *hilD*-overexpressing strain to myricanol was significantly reduced at 100 μM, while an excess of myricanol in the range of 100 to 400 μM, decreased the concentrations of the effector proteins in a dose-dependent manner (Supplementary Figure S5). We hypothesized that myricanol might interfere with the DNA binding ability of HilD. To test this speculation, we performed an electrophoretic mobility shift assay (EMSA). The DNA-binding ability of HilD was investigated using FAM-labeled *hilA* and *invF* promoters. The EMSA results suggest that HilD protein does bind specifically to the *hilA* and *invF* promoters as described (Akbar et al., 2003; Bustamante et al., 2008). However, the DNA-binding activity of HilD gradually decreased with dosage increase of myricanol in the gel shift reactions (Figure 5A). The EMSA results suggest therefore that myricanol is efficiently interfering with the DNA-binding ability of HilD.

To determine whether myricanol directly interacts with HilD, a thermal shift assay (TSA) was used. Following 1 h incubation with myricanol, the HilD was divided into equal parts and each aliquot was incubated at temperatures ranging from 44°C to 62°C for 3 min. The soluble proteins were subjected to 10% SDS-PAGE and then Coomassie-stained. The bands of HilD were more intense in the samples treated with myricanol compared to the negative control or treatment with DMSO. These results suggest that the stability of HilD can be strongly increased by treatment with myricanol (Figure 5B).

We then performed a DARTS assay to confirm that HilD was a molecular target of myricanol. S-tagged full-length HilD protein was expressed with 0.2% L-arabinose in *S.* Typhimurium, and the bacterial cell lysate was incubated with myricanol in *vitro*, followed by pronase E digestion. Western blot assay results suggested that the protease susceptibility of HilD was reduced following treatment with myricanol (Figure 5C). A central player in the invasion regulatory pathway is the HilA protein, which is a transcriptional activator belonging to the OmpR/ToxR family. Here, we chose HilA as a negative control (Supplementary Figure S6). Furthermore, we analyzed the binding of myricanol to HilD using a microscale thermophoresis (MST) assay. The data yielded a dissociation constant K*_*d*_* = 1.38 ± 0.53 μM for the interaction between myricanol and HilD (Figure 5D).

## Discussion

In the face of increasing antibiotic resistance, the targeting of bacterial virulence factors rather than bacterial survivability provides an alternative approach for the development of new antimicrobials. In this study, we first illustrated that myricanol (**1**) from *M. nagi* is an effective T3SS inhibitor and demonstrated its mechanism of action against *S.* Typhimurium T3SS. Importantly, myricanol did not affect bacterial growth or SW480 cell viability. Moreover, myricanol effectively prevented the secretion of the SPI-1 effectors and the invasion of *S.* Typhimurium into host cells. Those effects appear to be due to myricanol interfering with the DNA binding ability of HilD.

Medicinal plants are an attractive source of anti-virulence agents, and we are only beginning to see the real value of medicinal flora. The traditional uses of medicinal plants can give hints as to the activity of their chemical components (Abreu et al., 2012; Silva et al., 2016). Plants use various secondary metabolites to resist the attack of pathogens. T3SS serves as a primary anti-host defense mechanism for many gram-negative plant pathogens, such as the *Arabidopsis* defense compound sulforaphane (SFN) that functions primarily by inhibiting *Pseudomonas syringae* T3SS genes (Wang et al., 2020). Here, we speculate that *M. nagi* uses its secondary metabolites to protect itself from phytopathogens. Therefore, the T3SS-targeted approach used in searching for novel anti-virulence compounds appears to be highly promising compared with random screening strategies.

The study of vital targeted proteins in pathogenic bacteria is indispensable for the optimization of active molecules, and the mechanisms of T3SS inhibitors are different from those of conventional antibiotics. In this study, the MST results showed that myricanol could directly interact with HilD with a dissociation constant K*_*d*_* = 1.38 ± 0.53 μM. Myricanol is expected to serve as a probe to study the function and structure of HilD in the future. HilD is a member of the AraC/XylS family of transcriptional regulators, all consisting of two domains. The non-conserved domain seems to be involved in effector/signal recognition and dimerization. The conserved domain is characterized by significant amino acid sequence homology containing the DNA-binding domain of the family members (Gallegos et al., 1997; Tobes and Ramos, 2002). The stable dimer of HilD is likely to be related to the non-conserved region close to the N-terminal, and the DNA-binding domain close to the C-terminal also possesses two HTH motifs similar to MarA and Rob, two AraC/XylS family members where the crystal structures have been determined (Rhee et al., 1998; Kwon et al., 2000). If progress can be made in the elucidation of the crystal structure of HilD, future studies should focus on the binding domain of myricanol to HilD.

As an essential virulence factor, T3SS is highly conserved in structure and function among different gram-negative bacteria (Marlovits and Stebbins, 2009; Worrall et al., 2011; Diepold and Wagner, 2014). The AraC-XylS family of transcription proteins are also conserved in function and are broadly distributed through bacteria, and include LcrF/VirF in human pathogenic yersiniae, MxiE in *Shigella flexneri* and ExsA in *Pseudomonas aeruginosa* (Francis et al., 2002). These proteins are promising possible targets for the design of a broad spectrum of applications in anti-virulence. In this study, our identification of a cyclic diarylheptanoid compound myricanol isolated from the medicinal plant *M. nagi* has enriched the portfolio of anti-T3SS agents. Myricanol is a promising candidate for the development of novel anti-virulence agents.

## Data Availability Statement

The original contributions presented in the study are publicly available. This data can be found here: https://www.ncbi.nlm.nih.gov/, PRJNA639098.

## Author Contributions

YW: investigation and writing – original draft preparation. XY and DZ: resources. CL: writing – review and editing and supervision. All authors have read and agreed to the published version of the manuscript.

## Conflict of Interest

The authors declare that the research was conducted in the absence of any commercial or financial relationships that could be construed as a potential conflict of interest.

## Footnotes

1 https://www.cusabio.com/
