# Supplementary material for: Myricanol Inhibits the Type III Secretion System of Salmonella enterica Serovar Typhimurium by Interfering With the DNA-Binding Activity of HilD
Source: Front Microbiol. 2020 Sep 25;11:571217. doi: 10.3389/fmicb.2020.571217 (PMC7546796; doi:10.3389/fmicb.2020.571217)
Supplement: Supplementary file 1 [file Data_Sheet_1.PDF]

Supplementary Material

List of Contents

I. Supplementary tables for methods

Table S1. Strains and plasmids
Table S2. Primers pairs for real-time PCR or PCR

II. Supplementary results

Table S3. Differentially expressed genes involved in *Salmonella* infection.
Figure S1. Myricanol and **3** did not affect the growth of *S.* Typhimurium.
Figure S2. Secretion of effector proteins was inhibited by myricanol (**1**) and **3**.
Figure S3. Cytotoxicity assays of myricanol and compound **3**.
Figure S4. Myricanol did not induce the degradation of SipC.
Figure S5. Reduced sensitivity of *hilD*-overexpressed strain to myricanol.
Figure S6. There was no interaction between myricanol and HilA.

III. Supplementary NMR spectrum

Figure S7. ^{1}H NMR spectra of myricanol (**1**).
Figure S8. ^{13}C NMR spectra of myricanol (**1**).
Figure S9. ^{1}H NMR spectra of **2**.
Figure S10. ^{13}C NMR spectra of **2**.
Figure S11. ^{1}H NMR spectra of **3**.
Figure S12. ^{13}C NMR spectra of **3**.
Figure S13. ^{1}H NMR spectra of **4**.
Figure S14. ^{13}C NMR spectra of **4**.
Figure S15. ^{1}H NMR spectra of **5**.
Figure S16. ^{13}C NMR spectra of **5**.
Figure S17. ^{1}H NMR spectra of **6**.
Figure S18. ^{13}C NMR spectra of **6**.
Figure S20. ^{13}C NMR spectra of **7**.
Figure S21. ^{1}H NMR spectra of **8**.
Figure S22. ^{13}C NMR spectra of **8**.
Figure S23. ^{1}H NMR spectra of **9**.
Figure S24. ^{13}C NMR spectra of **9**.
Figure S25. ^{1}H NMR spectra of **10**.
Figure S26. ^{13}C NMR spectra of **10**.

I. Supplementary tables for methods

Table S1. Strains and plasmids used in this study.

Strains or plasmids	Description	Origin
**Strains**		
S. enterica serovar Typhimurium UK-1 χ8956	△PrpoS183::TT araC pBAD *rpoS*	(Curtiss et al. 2009)
BL21 (DE3)	F- *ompT gal dcm lon hsdSB* (rB- mB-) λ(DE3 [*lacI lacUV*5-T7 gene 1 *ind1 sam7 nin5*])	Invitrogen
E. coli DH5α	F- 80*lac*Z M15 (*lacZYA–argF*) U169*eoR recA1 endA1 hsdR17 phoA supE*44-thi-1 *gyrA96 relA1*	Invitrogen
**Plasmids**		
pBAD-*hilA*-pWSK29	pWSK29 carrying araC pBAD and *hilA*, Ap^r	(Li et al. 2013)
pBAD-*hilD-s*-pWSK29	pWSK29 carrying araC pBAD and *hilD*-S *tag*, Ap^r	This study
pET28a-*hilD*	pET28a carrying *hilD*, Kan^r	This study

Table S2. Primers pairs for real-time PCR or PCR

Primer name	Primer sequence
qPCR-*hilA*-F	catacattggcgatacttccttt
qPCR-*hilA*-R	gcatactgcgataatcccttca
qPCR-*invF*-F	ggcgcaggattagtggacac
qPCR-*invF*-R	acgatcttgccaaatagcgc
qPCR-*sicA*-F	ggcagcaaaagccagacagt
qPCR-*sicA*-R	cgcctccagatagaccaacg

qPCR-*sipC*-F	gtgacctggggttgagtcctac
qPCR-*sipC*-R	aaggacgtgatcgttccgg
qPCR-*prgH*-F	gttgtgggctcgtcaggttt
qPCR-*prgH*-R	cgcttattttcttcgttttcgt
hilA-promoter-F	5'FAM-ctctattgcaatgaggc
hilA-promoter-R	5'FAM-caggaacaggattaaaa
invF-promoter-F	5'FAM-agcagcgcgccttcctg
invF-promoter-R	5'FAM-gccgatcagtaaaaaga

II. Supplementary results

Table S3. Differentially expressed genes involved in *Salmonella* infection.

Gene ID	Name	Description	Log_2 fold change
STMUK_RS05365	*sopB*	*Salmonella* infection	-7.898779427
STMUK_RS14630	*invF*	AraC family transcriptional regulator	-6.945479673
STMUK_RS09275	*sopE*	Invasion of epithelial cells	-6.200551827
STMUK_RS14625	*invG*	Type III secretion system outer membrane ring protein	-6.012082932
STMUK_RS14500	*prgI*	Type III secretion system needle major subunit	-5.762400996
STMUK_RS05360	*sigE*	Type III secretion system chaperone	-5.676324362
STMUK_RS14505	*prgH*	Type III secretion system needle major subunit	-5.661581444
STMUK_RS14555	*sipC*	SPI-1 effector protein	-5.545870628

STMUK_RS14620	*invE*	Type III secretion system gatekeeper	-5.343405484
STMUK_RS14560	*sipB*	SPI-1 effector protein	-5.269941543
STMUK_RS14550	*sipD*	SPI-1 effector protein	-5.179205677
STMUK_RS10640	*sopA*	Secreted effector protein	-5.015971298
STMUK_RS14495	*prgJ*	Type III secretion system needle major subunit	-4.669348327
STMUK_RS14545	*sipA*	Actin-binding protein	-4.373558525
STMUK_RS14515	*hilA*	Invasion protein regulator	-4.320974603
STMUK_RS14490	*prgK*	Type III secretion system lipoprotein	-4.279184321
STMUK_RS14565	*sicA*	Type III secretion system chaperone	-4.01051264
STMUK_RS14610	*spaK*	Type III secretion system chaperone	-3.689589908
STMUK_RS14635	*invH*	invasion lipoprotein	-3.407389355
STMUK_RS14590	*spaO*	Type III secretion system protein	-3.053249735
STMUK_RS14595	*spaN*	Type III secretion system protein	-3.048139226
STMUK_RS14605	*spaL*	Type III secretion system ATPase	-2.888213849
STMUK_RS14860	*sopD*	Type III virulence effector protein	-2.748330518
STMUK_RS14585	*spaP*	Type III secretion system export apparatus protein	-2.721865146
STMUK_RS14520	*iagB*	Invasion protein	-2.661683911
STMUK_RS14600	*spaM*	Type III secretion system protein	-2.445664325
STMUK_RS14470	*hilC*	AraC family transcriptional regulator	-2.397897906
STMUK_RS14510	*hilD*	AraC family transcriptional regulator	-2.366970702

STMUK_RS14580	*spaQ*	Type III secretion system export apparatus protein	-2.114725855

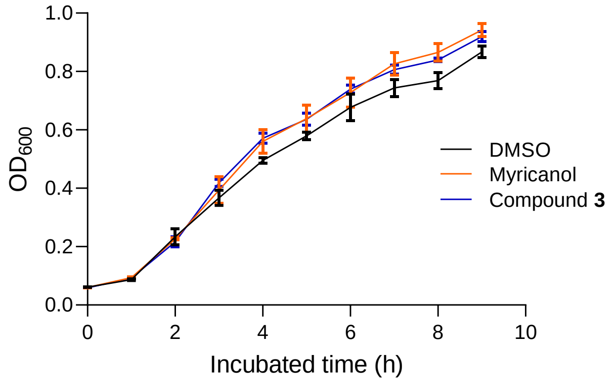

Figure S1. Myricanol and compound 3 did not affect the growth of *S.* Typhimurium. *S.* Typhimurium was grown under the treatment with 200 µM of myricanol (**1**), monomethylmyricanol (**3**) or an equivalent volume of DMSO, respectively. The OD_{600} of the culture was measured by a microplate reader (TECAN) every hour. Three replicates were measured in each experiment.

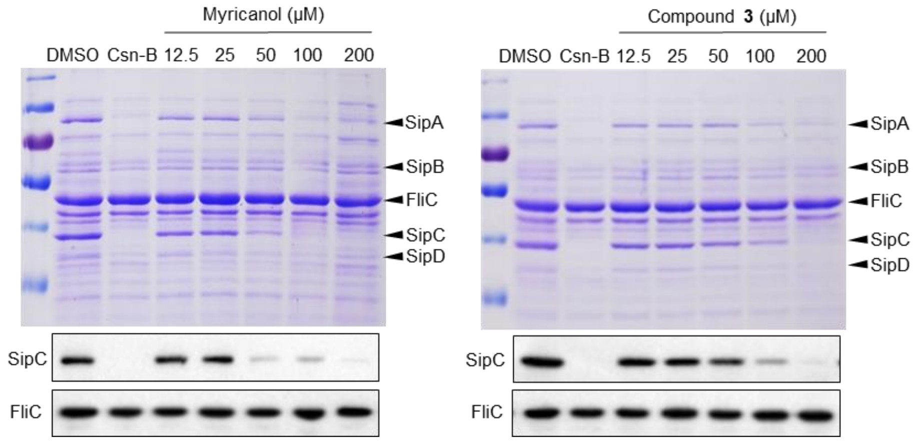

Figure S2. Myricanol (1) and monomethylmyricanol (3) inhibited the secretion of SPI-1 effector proteins in a dose-dependent manner. Supernatant protein samples were resolved on 10% SDS-PAGE and followed by Coomassie blue staining and or Western blotting.

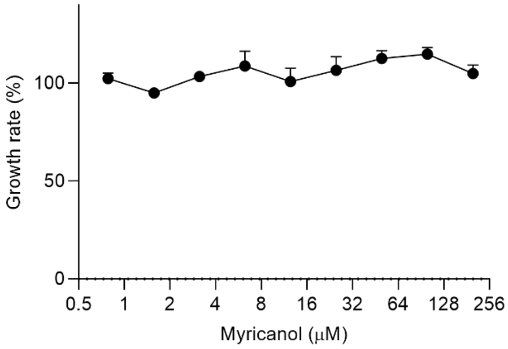

Figure S3. Myricanol did not affect the growth rate of SW480 cells. Cells were treated with different concentrations of myricanol for 12 h, 10% CCK-8 reagent was added in each well and the plates were incubated at 37 °C for 3.5 h. The cell viability was determined by measuring the value of OD_{450}.

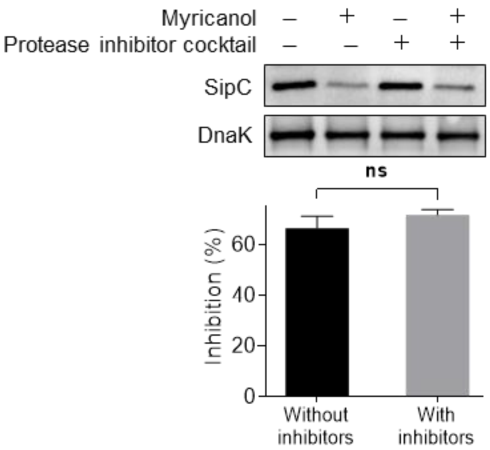

Figure S4. Myricanol did not induce the degradation of SipC. SipC protein levels in *S.* Typhimurium treated with myricanol in the absence or presence of protease inhibitor cocktail (Beyotime) determined by western blot. The reduction in SipC protein caused by myricanol treatment cannot be rescued by protease inhibitor cocktail, indicating that myricanol did not affect SipC protein stability.

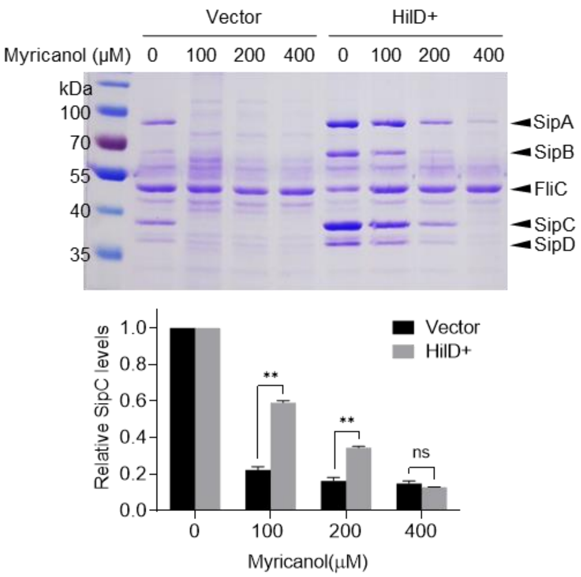

Figure S5. Reduced sensitivity of *hilD*-overexpressed strain to myricanol. In the *hilD*-overexpressed strain, the supernatant effector protein levels of SipA, SipB, SipC and SipD were all increased. And the sensitivity of *hilD*-overexpressed strain to myricanol was significantly reduced at 100 μM. **, P < 0.01.

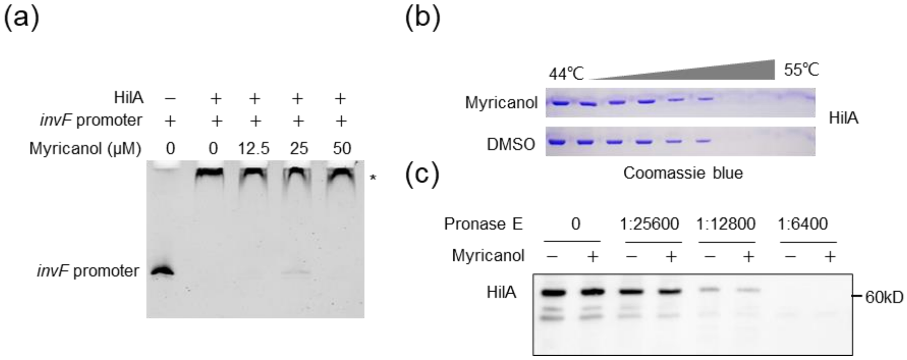

Figure S6. There was no interaction between myricanol and HilA. A central player in the invasion regulatory pathway is the HilA protein, which is a transcriptional activator belonging to the OmpR/ToxR family. (**a**) The DNA-binding ability of HilA was investigated using FAM-labeled invF promoter. This finding indicated that the myricanol does not interfere with the DNA-binding activity of HilA. (**b**) Myricanol (50 μM) and HilA protein were mixed in an equimolar ratio, and the mixture was incubated for 1 h at room temperature. Then the samples were subjected to thermal shift assay (TSA). The results showed that the thermal stability of HilA cannot be increased by the treatment of myricanol, compared to control. (**c**) In the DARTS assay, pre-incubated myricanol (50 μM) also cannot reduce the protease susceptibility of HilA in Salmonella lysate

III. Supplementary NMR spectrum

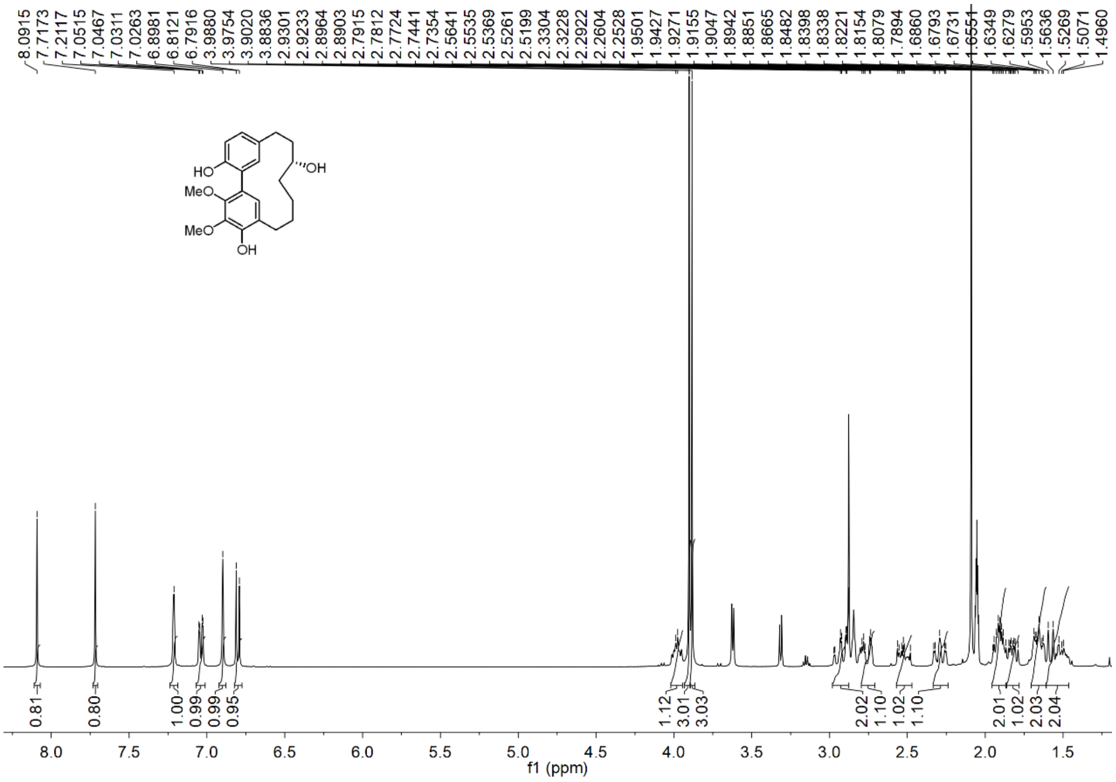

Figure S7. ^{1}H NMR spectra of myricanol (**1**) in acetone-d_6

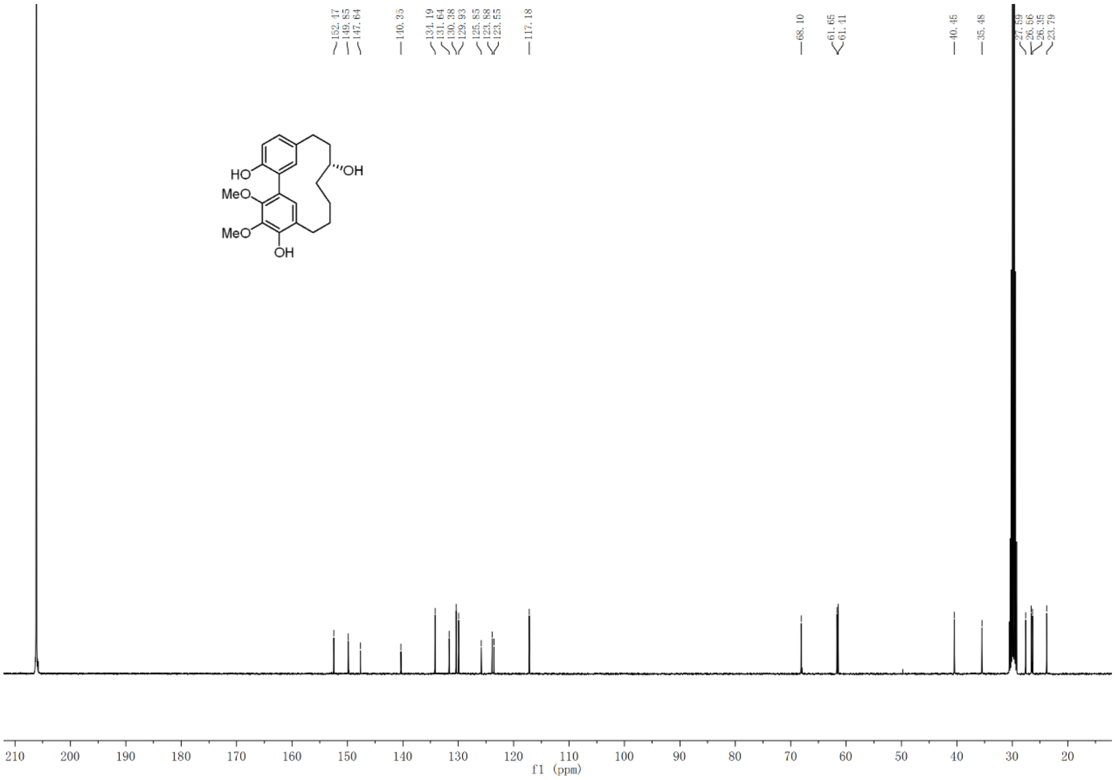

Figure S8 ^{13}C NMR spectra of myricanol (**1**) in acetone-d_6

(*R*)-1^5,1^6-dimethoxy-1,2(1,3)-dibenzenacyclononaphane-1^4,2^6,5-triol(Myricanol, 1)(Martin et al. 2015). ^{1}H NMR (400 MHz, acetone-d_6): δ 8.09 (1H, s, OH), 7.71 (1H, s, OH), 7.21 (1H, d, *J* = 2.0, H-18), 7.05 (1H, dd, *J* = 8.2, 2.0 Hz, H-15), 6.90 (1H, s, H-19), 6.81 (1H, *J* = 8.2 Hz, H-16), 4.01 (1H, m, H-11), 3.90 (3H, s, 4-OMe), 3.88 (3H, s, 3-OMe), 2.85 - 2.97 (2H, m, H-13), 2.79 (1H, dt, *J* = 18, 2.4 Hz, H-7a), 2.56 (1H, m, H-7b), 2.33 (1H, m, H-12b), 1.76-1.96 (3H, m, H-8, H-10b), 1.60-1.70 (2H, m, H-9b, H-12a), 1.45-1.60 (2H, m, H-9a, H-10a); ^{13}C NMR (100 MHz, acetone-d_6): δ 152.5(C-17), 149.9(C-5), 147.6 (C-3), 140.4 (C-4), 134.2 (C-18), 131.6 (C-14), 130.4 (C-15), 129.9 (C-19), 125.9 (C-1), 123.9 (C-2), 123.6 (C-6), 117.2 (C-16), 68.1 (C-11), 61.7 (3-OMe), 61.4 (4-OMe), 40.5(C-10), 35.5(C-12), 27.6 (C-13), 26.6 (C-8), 26.4 (C-7), 23.8 (C-9). $[\alpha]_D^{25}$ = -9.8 (*c* = 0.1, MeOH), ESI-MS: *m/z* 359.2 $[M + H]^+$.

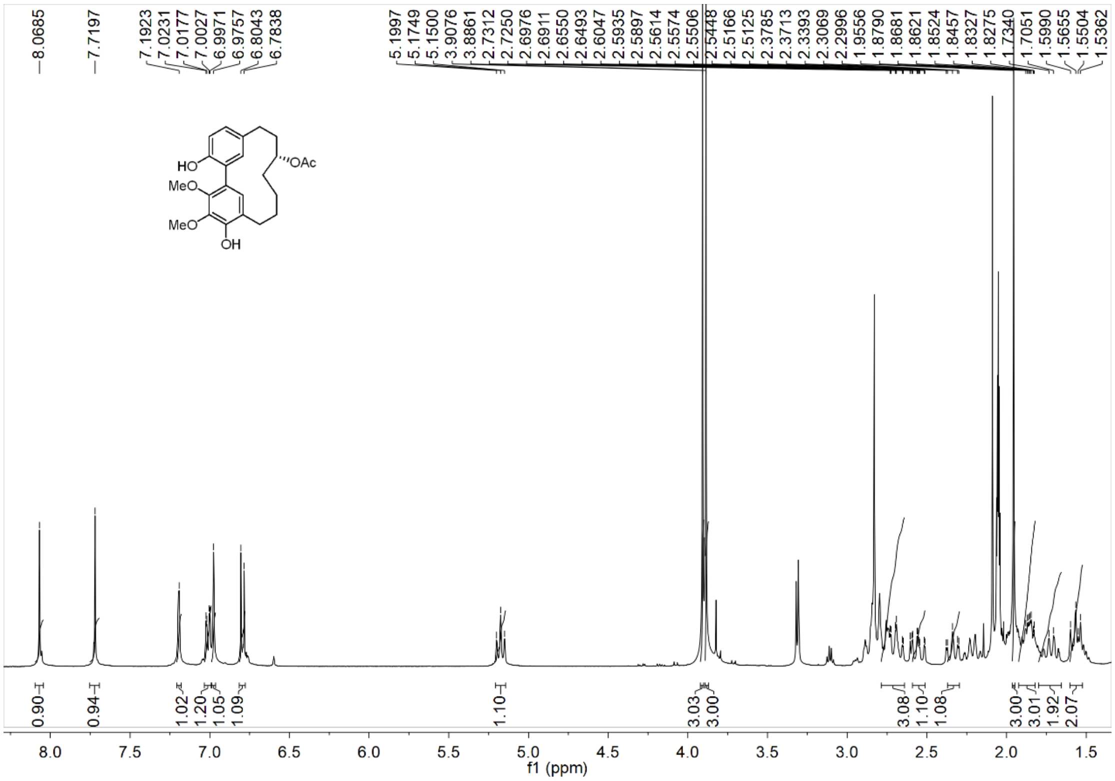

Figure S9. ^{1}H NMR spectra of **2** in acetone-d_6

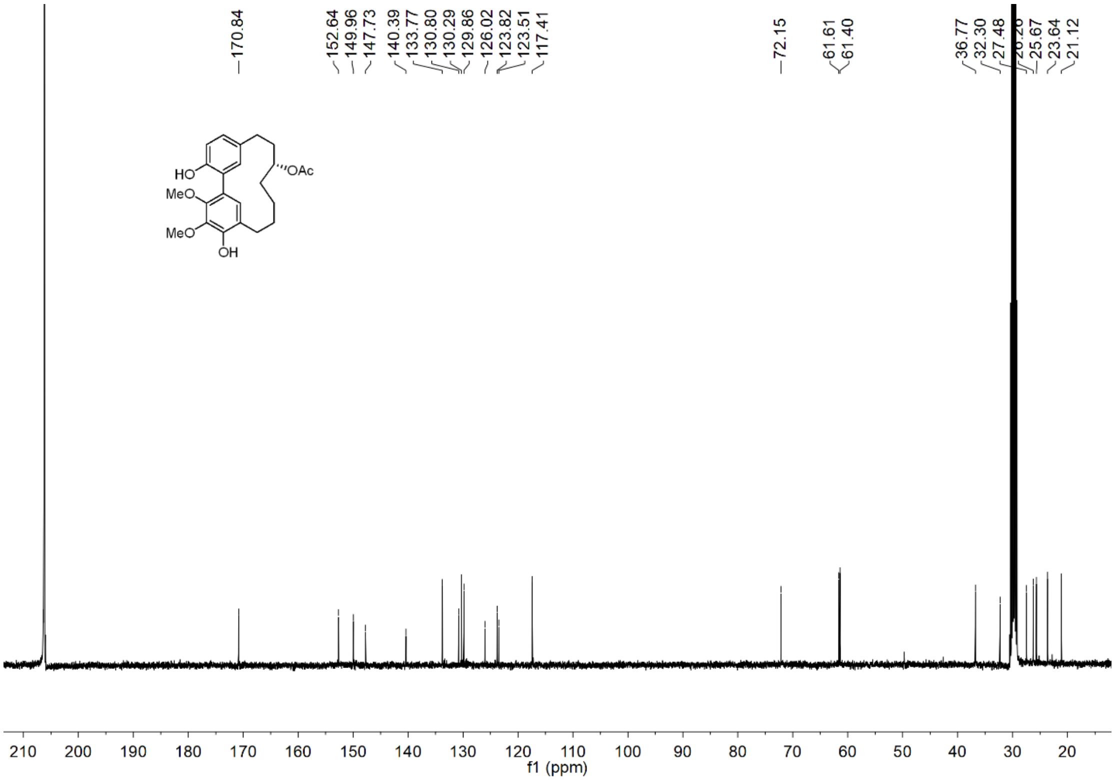

Figure S10. ^{13}C NMR spectra of **2** in acetone-d_6

***(R)*-1^4,2^6-dihydroxy-1^5,1^6-dimethoxy-1,2(1,3)-dibenzenacyclononaphane-5-yl acetate**(**2**) (Whiting and Wood 1980). ^{1}H NMR (400 MHz, acetone-d_6): δ 8.07 (1H, s, OH), 7.72 (1H, s, OH), 7.20 (1H, d, *J* = 2.0, H-18), 7.02 (1H, dd, *J* = 8.2, 2.0 Hz, H-15), 6.98 (1H, s, H-19), 6.80 (1H, *J* = 8.2 Hz, H-16), 5.20 (1H, m, H-11), 3.90 (3H, s, 4-OMe), 3.88 (3H, s, 3-OMe), 2.64-2.78 (3H, m, H-13, H-7a), 2.58 (1H, m, H-7b), 2.38 (1H, m, H-12b), 1.96 (3H, s, H-11-2$^'$), 1.82-1.92 (3H, m, H-8, H-10b), 1.65-1.79 (2H, m, H-9b, H-12a), 1.53-1.60 (2H, m, H-9a, H-10a); ^{13}C NMR (100 MHz, acetone-d_6) δ 170.8 (11-1$^'$), 152.6 (C-17), 150.0 (C-5), 147.7 (C-3), 140.4 (C-4), 133.8 (C-18), 130.8 (C-14), 130.3 (C-15), 129.9 (C-19), 126.0 (C-1), 123.8 (C-2), 123.5 (C-6), 117.4 (C-16), 72.2 (C-11), 61.6 (3-OMe), 61.4 (4-OMe), 36.8 (C-10), 32.3 (C-12), 27.5 (C-13), 26.3 (C-8), 25. 7(C-7), 23.6 (C-9), 21.1 (11-2$^'$). $[\alpha]_D^{25}$ = -15.3 (*c* = 0.1, MeOH), ESI-MS: *m/z* 401.2 [M + H]$^+$.

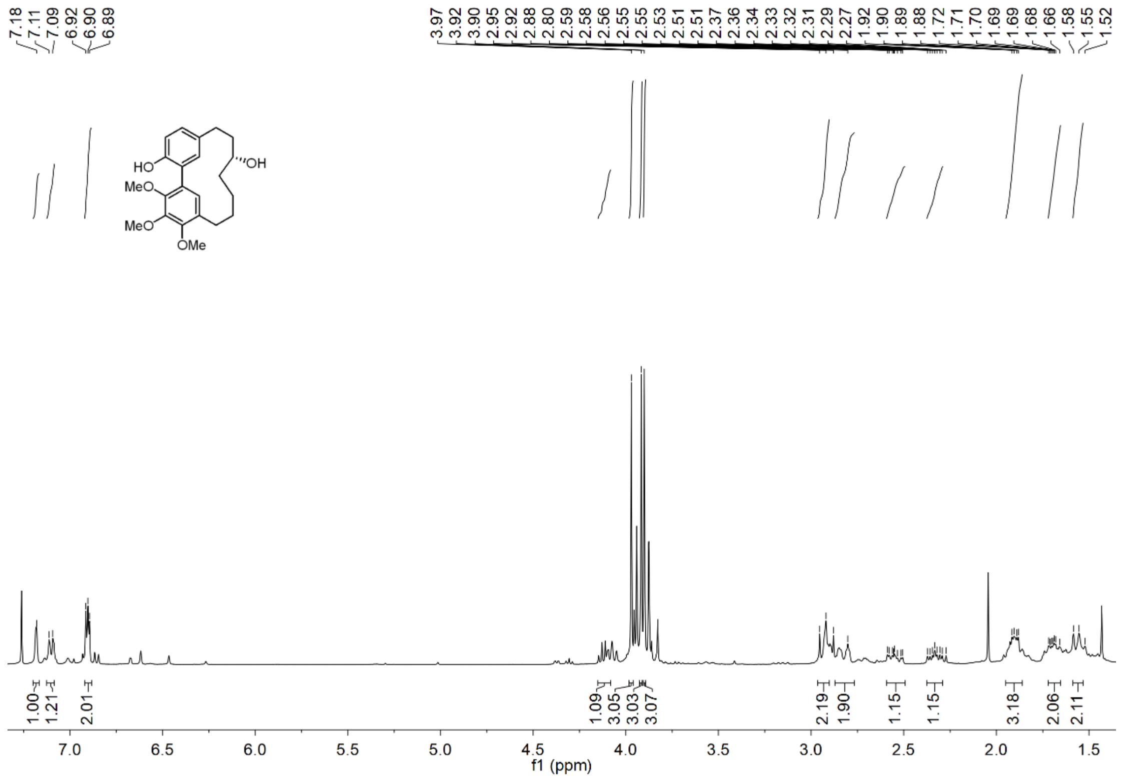

Figure S11. ^{1}H NMR spectra of **3** in $CDCl_3$

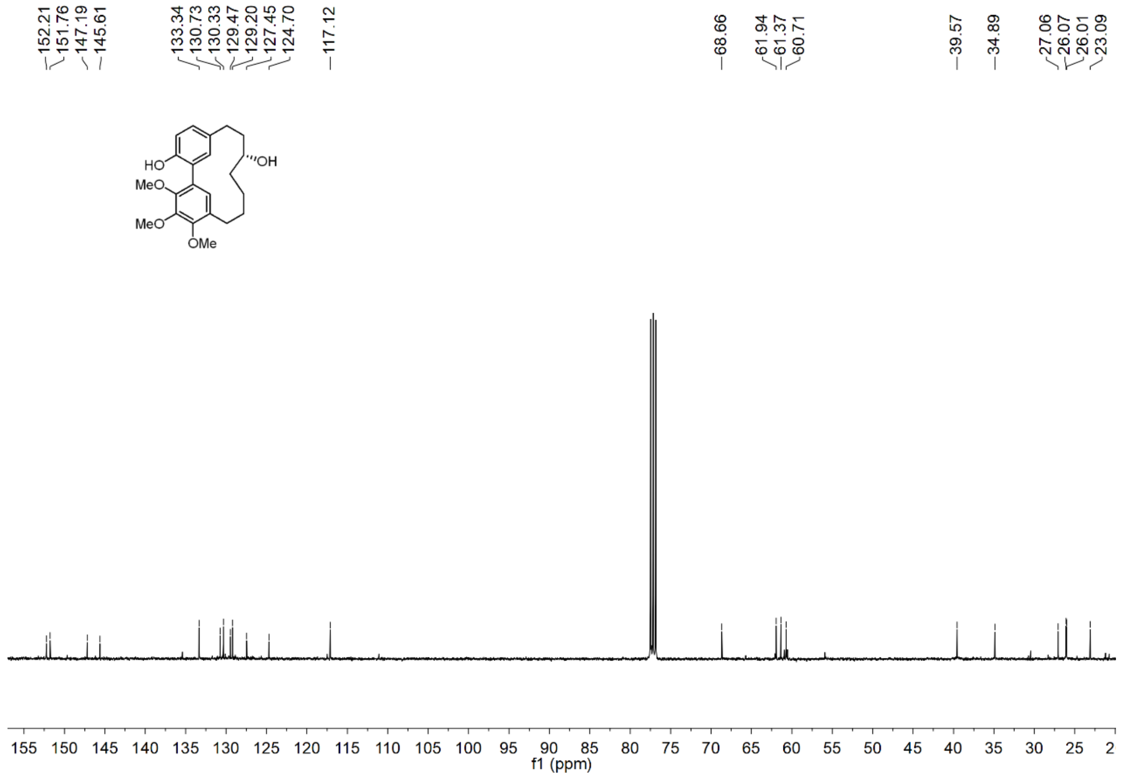

Figure S12. ^{13}C NMR spectra of **3** in $CDCl_3$

(*R*)-2^4,2^5,2^6-trimethoxy-1,2(1,3)-dibenzenacyclononaphane-16,7-diol, (Monomethylmyricanol, 3) (Begley et al. 1971). ^{1}H NMR (400 MHz, $CDCl_3$): δ 7.26 (1H, s, OH), 7.18 (1H, d, *J* = 2.0, H-18), 7.11 (1H, dd, *J* = 8.2, 2.0 Hz, H-15), 6.92 (2H, m, H-16, H-19), 4.15 (1H, m, H-11), 3.97 (3H, s, 4-OMe), 3.92 (3H, s, 5-OMe), 3.90 (3H, s, 3-OMe), 2.95 - 2.88 (2H, m, H-13), 2.87 (1H, dt, *J* = 18, 2.4 Hz, H-7a), 2.60 (1H, m, H-7b), 2.37 (1H, m, H-12b), 1.96 (3H, s, H-11-2'), 1.89-1.94 (3H, m, H-8, H-10b), 1.66-1.72 (2H, m, H-9b, H-12a), 1.55-1.58 (2H, m, H-9a, H-10a); ^{13}C NMR (100MHz, $CDCl_3$) δ 152.2 (C-17), 151.8 (C-5), 147.2 (C-3), 145.6 (C-4), 133.3(C-18), 130.7 (C-14), 130.3 (C-15), 129.5 (C-19), 129.2 (C-1), 127.5(C-2), 124.7 (C-6), 117.1 (C-16), 68.7 (C-11), 61.9 (3-OMe), 61.4 (4-OMe), 60.7 (5-OMe), 39.6 (C-10), 34.9 (C-12), 27.1 (C-13), 26.1 (C-7), 26.0 (C-8), 23.1 (C-9). $[\alpha]_D^{25}$ = -11.8 (c = 0.1, MeOH), ESI-MS: *m/z* 373.2 $[M + H]^+$.

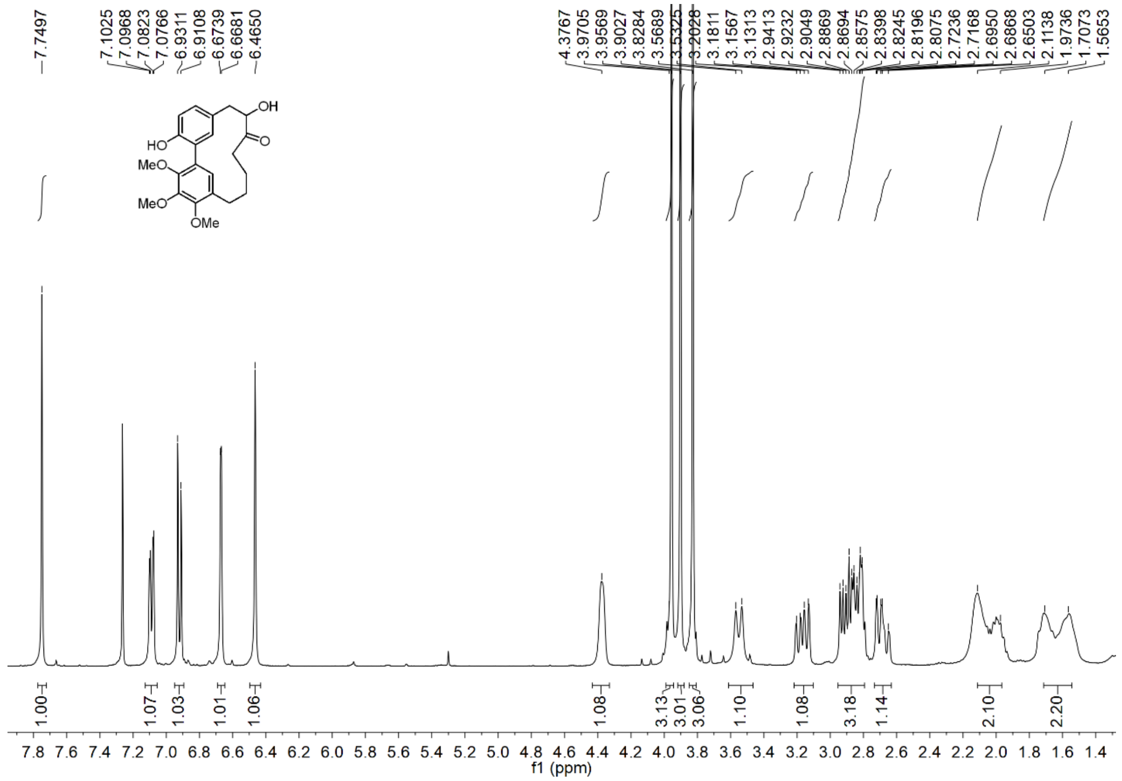

Figure S13. ^{1}H NMR spectra of **4** in $CDCl_3$

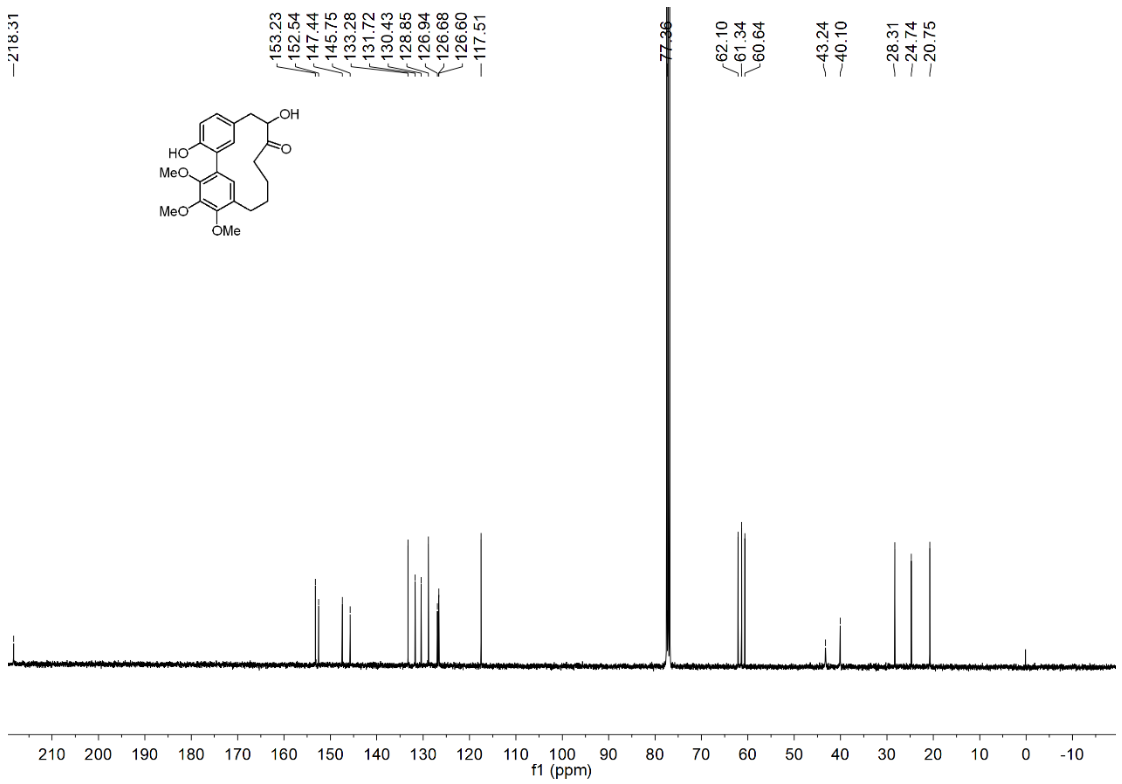

Figure S14. ^{13}C NMR spectra of **4** in $CDCl_3$

2^6,4-dihydroxy-1^4,1^5,1^6-trimethoxy-1,2(1,3)-dibenzenacyclononaphan-5-one (Porson, 4)(Nagai et al. 1995)**.** ^{1}H NMR (400 MHz, $CDCl_3$): δ 7.75 (1H, s, OH), 7.10 (1H, dd, *J*= 8.2, 2.0 Hz, H-15), 6.93 (1H, d, *J*= 8.2 Hz, H-16), 6.67 (1H, d, *J*= 2.0, H-18), 6.47 (1H, s, H-19), 4.38 (1H, d, *J*= 5.6 Hz, H-12), 3.97 (3H, s, 4-OMe), 3.95 (3H, s, 5-OMe), 3.90 (3H, s, 3-OMe), 3.56 (1H, d, *J*= 14.4 Hz, H-13a), 3.20 (1H, dd, *J*= 18.4, 8.7 Hz, H-10a), 2.81-2.94 (3H, m, H-13b, H-10b, H-7a), 2.72 (1H, m, H-7b), 1.97-2.11 (2H, m, H-8a, H-9a), 1.56-1.70 (2H, m, H-8b, H-9b); ^{13}C NMR (100 MHz, $CDCl_3$) δ 218.3 (C-11), 153.2 (C-17), 152.5 (C-5), 147.4 (C-3), 145.8 (C-4), 133.3 (C-18), 131.7 (C-14), 130.4 (C-15), 128.9 (C-19), 126.9 (C-1), 126.7 (C-2), 126.6 (C-6), 117.5 (C-16), 77.4 (C-12), 62.1 (3-OMe), 61.3 (4-OMe), 60.6 (5-OMe), 43.2 (C-10), 40.1 (C-13), 28.3 (C-7), 24.7 (C-8), 20.8 (C-9). ESI-MS: *m/z* 387.2 $[M + H]^+$.

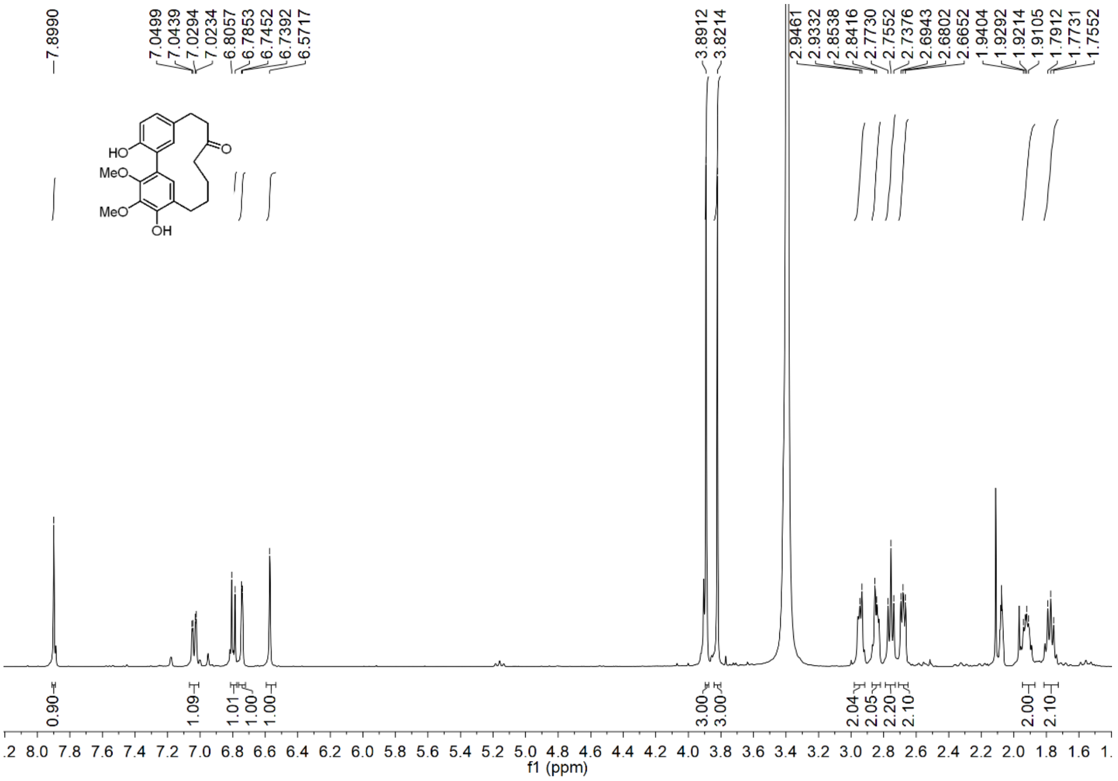

Figure S15. ^{1}H NMR spectra of **5** in acetone-d_6

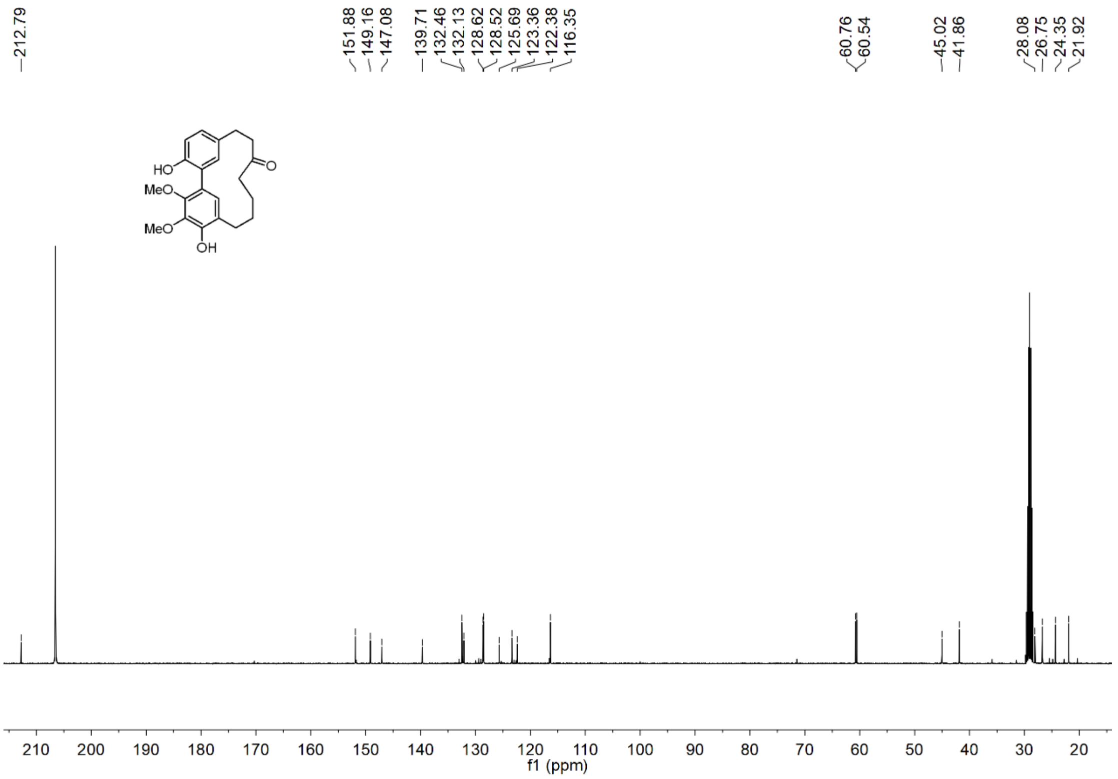

Figure S16 ^{13}C NMR spectra of **5** in acetone-d_6

1^4,2^6-dihydroxy-1^5,1^6-dimethoxy-1,2(1,3)-dibenzenacyclononaphan-5-one(**Myricanone, 5**) (Begley et al. 1971, Sun et al. 1988). ^{1}H NMR (400 MHz,acetone-d_6): δ 7.90 (1H, s, OH), 7.05 (1H, dd, *J*= 8.2, 2.4 Hz, H-15), 7.05 (1H, d, *J*= 8.2 Hz, H-16), 6.74 (1H, d, *J*= 2.4 Hz H-18), 6.57 (1H, s, H-19), 4.01 (1H, m, H-11), 3.89 (3H, s, 4-OMe), 3.82 (3H, s, 3-OMe), 2.95 (2H, m, H-13), 2.85 (2H, m, H-12), 2.77 (2H, m, H-10), 2.69 (2H, m, H-7), 1.94 (2H, m, H-8), 1.79 (2H, m, H-9); ^{13}C NMR (100 MHz, acetone-d_6): δ 212.8 (C-11), 151.9 (C-17), 149.2 (C-5), 147.1 (C-3), 139.7 (C-4), 132.5 (C-18), 132.1 (C-14), 128.6 (C-15), 128.5 (C-19), 125.7 (C-1), 123.4 (C-2), 122.4 (C-6), 116.4 (C-16), 60.8 (3-OMe), 60.5 (4-OMe), 45.0 (C-10), 41.9 (C-12), 28.1(C-13), 26.8 (C-7), 24.4 (C-8), 21.9 (C-9). ESI-MS: *m/z* 357.2 $[M + H]^+$.

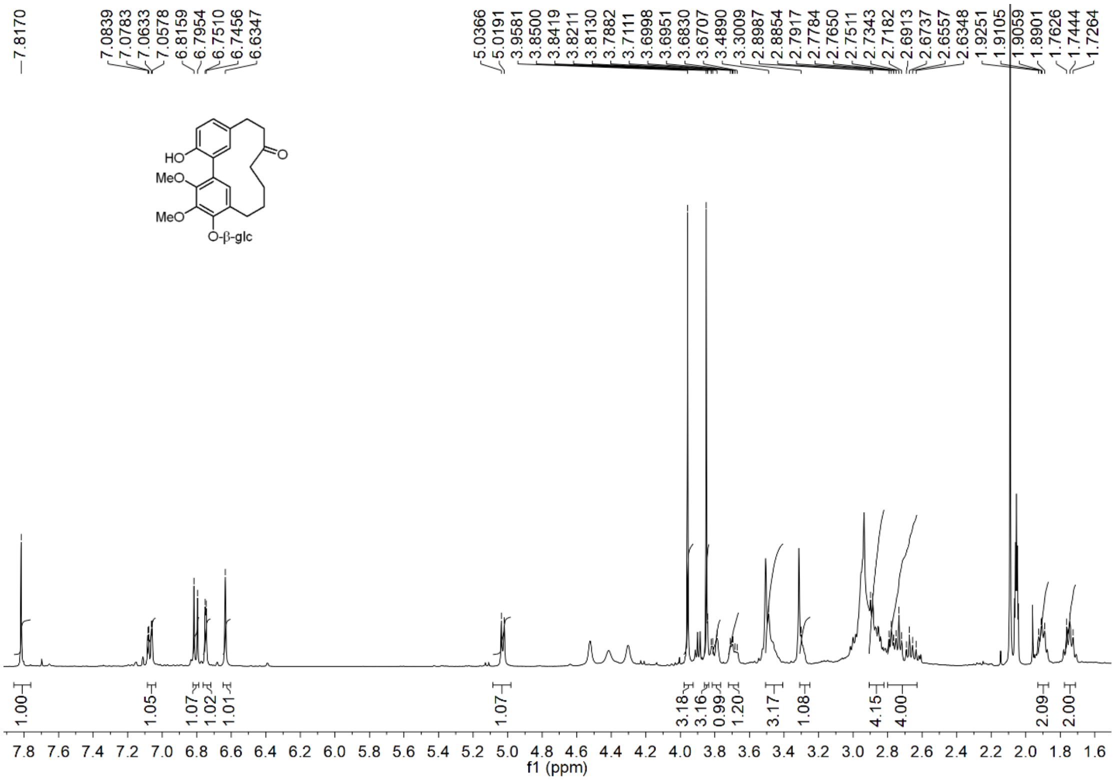

Figure S17. ^{1}H NMR spectra of **6** in acetone-d_6

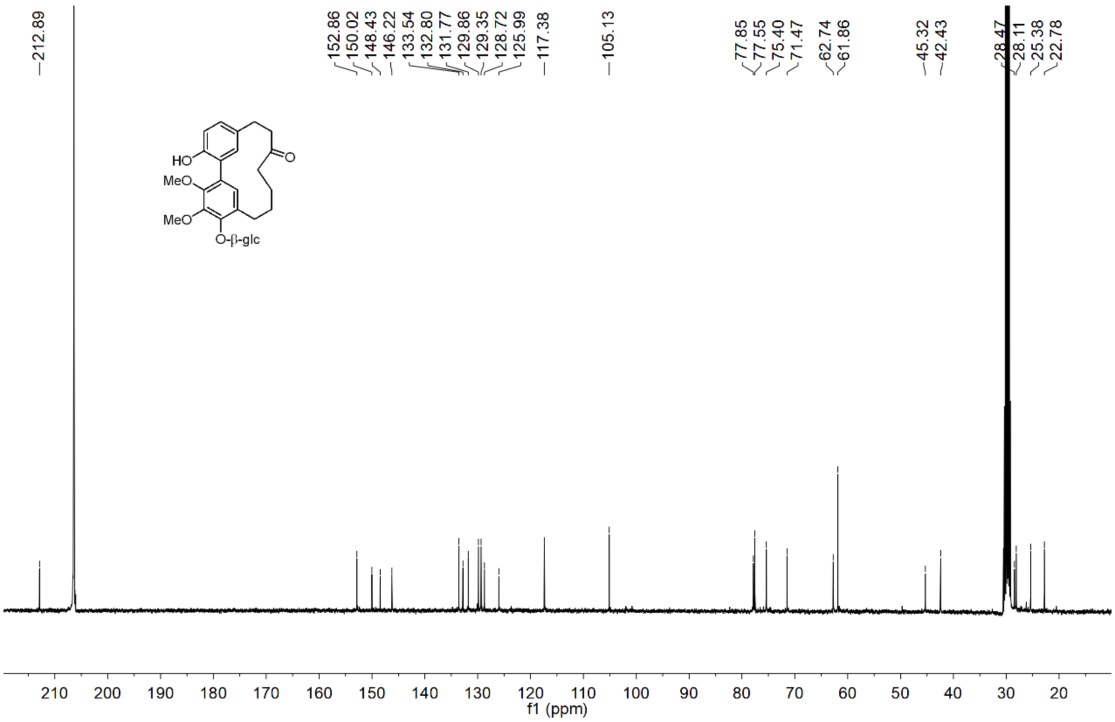

Figure S18 ^{13}C NMR spectra of **6** in acetone-d_6

2^6-hydroxy-1^5,1^6-dimethoxy-1^4-(((4*S*,5*S*,6*S*)-3,4,5-trihydroxy-6-(hydroxymethyl)tetrahydro-2H-pyran-2-yl)oxy)-1,2(1,3)-dibenzenacyclononaphan-5-one (Myricanone 5-*O*-β-D-glucopyranoside, 6)(Tene et al. 2000)**.** ^{1}H NMR (400 MHz, acetone-d_6): δ 7.81 (1H, s, OH), 7.08 (1H, dd, *J* = 8.2, 2.4 Hz, H-15), 6.82 (1H, d, *J* = 8.2 Hz, H-16), 6.75 (1H, d, *J* = 2.4 Hz H-18), 6.63 (1H, s, H-19), 5.04 (1H, d, *J*= 7 Hz, H-5-1'), 4.01 (1H, m, H-11), 3.89 (3H, s, 4-OMe), 3.82 (3H, s, 3-OMe), 3.82 (1H, m, H-6'a), 3.70 (1H, m, H-6'b), 3.39-3.48 (3H, m, H-2', 3',4'), 3.30 (1H, m, H-5'), 2.82-2.90 (4H, m, H-12, H-13), 2.63-2.79 (4H, m, H-7, H-10), 1.92 (2H, m, H-8), 1.76 (2H, m, H-9); ^{13}C NMR (100 MHz, acetone-d_6) δ 212.9 (C-11), 152.9 (C-17), 150.0 (C-5), 148.4 (C-3), 146.2 (C-4), 133.5 (C-18), 132.8 (C-14), 131.8 (C-15), 129.9 (C-19), 129.4 (C-1), 128.7 (C-2), 126.0 (C-6), 117.4 (C-16), 105.1 (C-1'), 77.9 (C-5'), 77.6 (C-3'), 75.4 (C-2'), 71.5 (C-4'), 62.7 (C-6'), 61.9 (3,4-OMe), 45.3 (C-10), 42.4 (C-12), 28.5 (C-13), 28.1 (C-7), 25.4 (C-8), 22. 8 (C-9). ESI-MS: *m/z* 519.2 [M + H]$^+$.

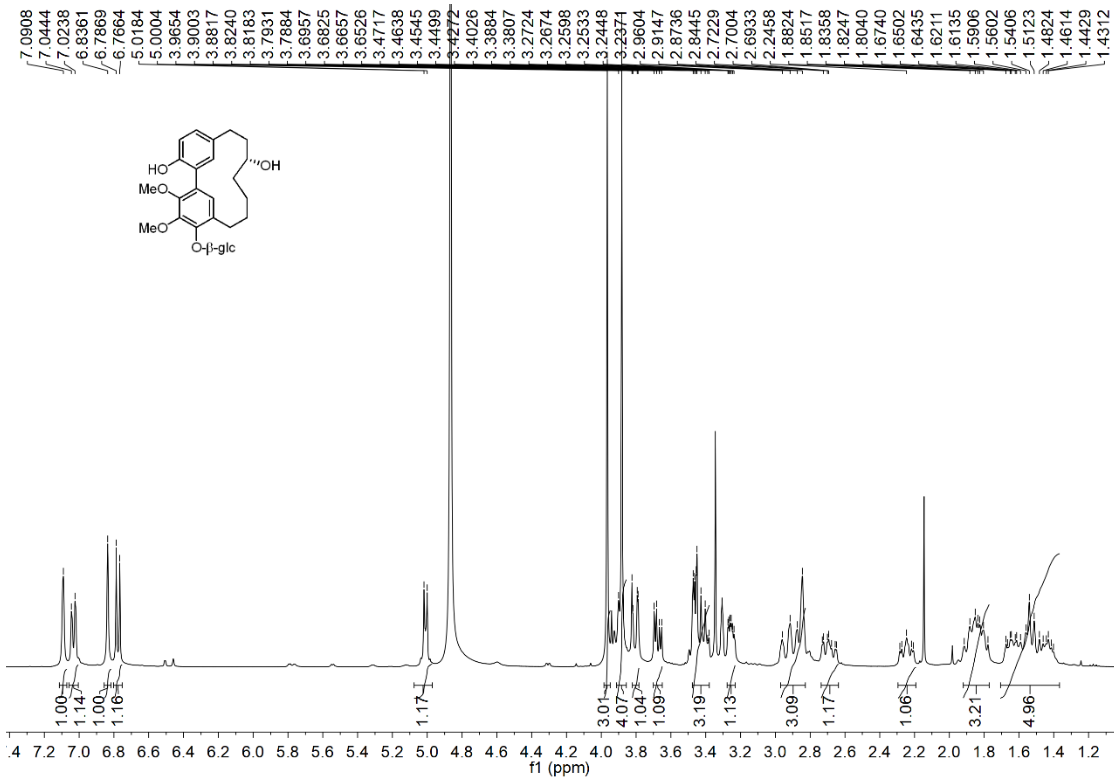

Figure S19. ^{1}H NMR spectra of **7** in CD_3OD

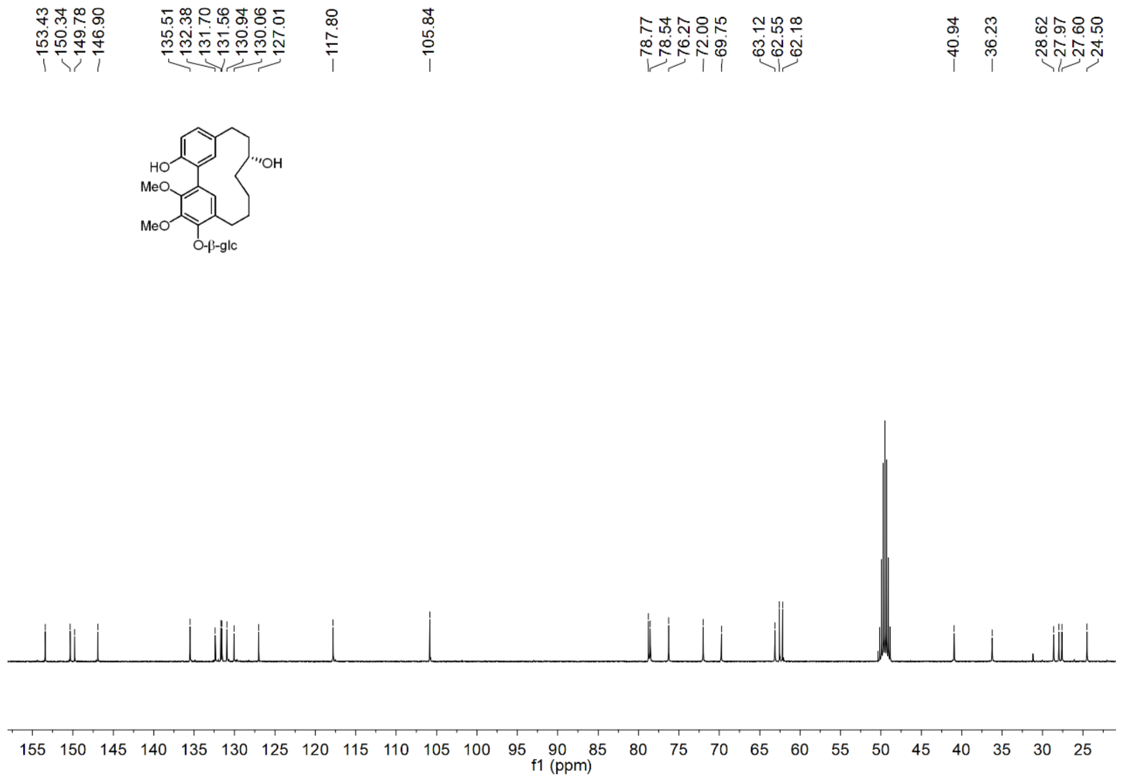

Figure S20 ^{13}C NMR spectra of **7** in CD_3OD

(4*S*,5*S*,6*S*)-2-(((*R*)-2^6,5-dihydroxy-1^5,1^6-dimethoxy-1,2(1,3)-dibenzenacyclononaphane-14-yl)oxy)-6-(hydroxymethyl)tetrahydro-2H-pyran-3,4,5-triol, 7)(Inoue, Arai, and Nagai 1984). ^{1}H NMR (400 MHz, CD_3OD): δ 7.09 (1H, d, *J*= 2.4 Hz H-18), 7.04 (1H, dd, *J*= 8.2, 2.4 Hz, H-15), 6.84 (1H, s, H-19), 6.79 (1H, d, *J*= 8.2 Hz, H-16), 5.04 (1H, d, *J*= 7, H-1'), 3.97 (3H, s, 4-OMe), 3.85-3.90 (4H, m, 3-OMe, H-11), 3.82 (1H, dd, *J*= 2.28, 12.4 Hz, H-6'a), 3.70 (1H, dd, *J*= 5.3, 12.0 Hz, H-6'b), 3.38-3.47 (3H, m, H-2', 3',4'), 3.27 (1H, m, H-5'), 2.84-3.96 (3H, m, H-13, H-7a), 2.73-2.65 (1H, m, H-7b), 2.20-2.28 (1H, m, H-12a), 1.77-1.91 (3H, m ,H-10a, H-8), 1.40-1.67 (5H, m, H-12b, H-10b, H-9, 11-OH); ^{13}C NMR (100 MHz, CD_3OD): δ 153.4 (C-17), 150.3 (C-4), 149.8 (C-5), 146.9 (C-3), 135.5 (C-18), 132.4 (C-14), 131.7 (C-6), 131.6 (C-14), 130.9 (C-19), 130.1 (C-2), 127.0 (C-1), 117.8 (C-16), 105.8 (C-1'), 78.8 (C-5'), 78.5 (C-3'), 76.3 (C-2'), 72.0 (C-4'), 69.8(C-11), 63.1 (C-6'), 62.6 (4-OMe), 62.2(3-OMe), 40.9 (C-10), 36.2 (C-12), 28.6 (C13), 28.0(C-7), 27.6 (C-8), 24.5 (C-9). $[\alpha]_D^{25}$ = -19.3 (*c* = 0.1, MeOH), ESI-MS: *m/z* 521.2 $[M + H]^+$.

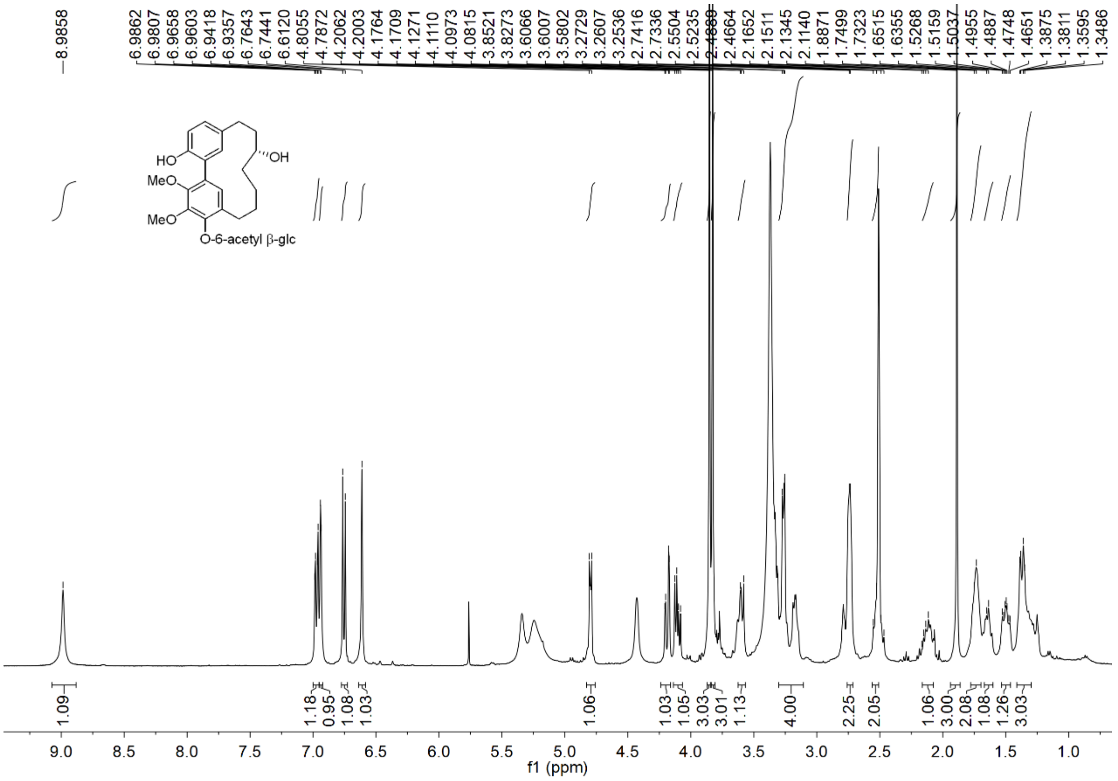

Figure S21. ^{1}H NMR spectra of **8** in CD_3OD

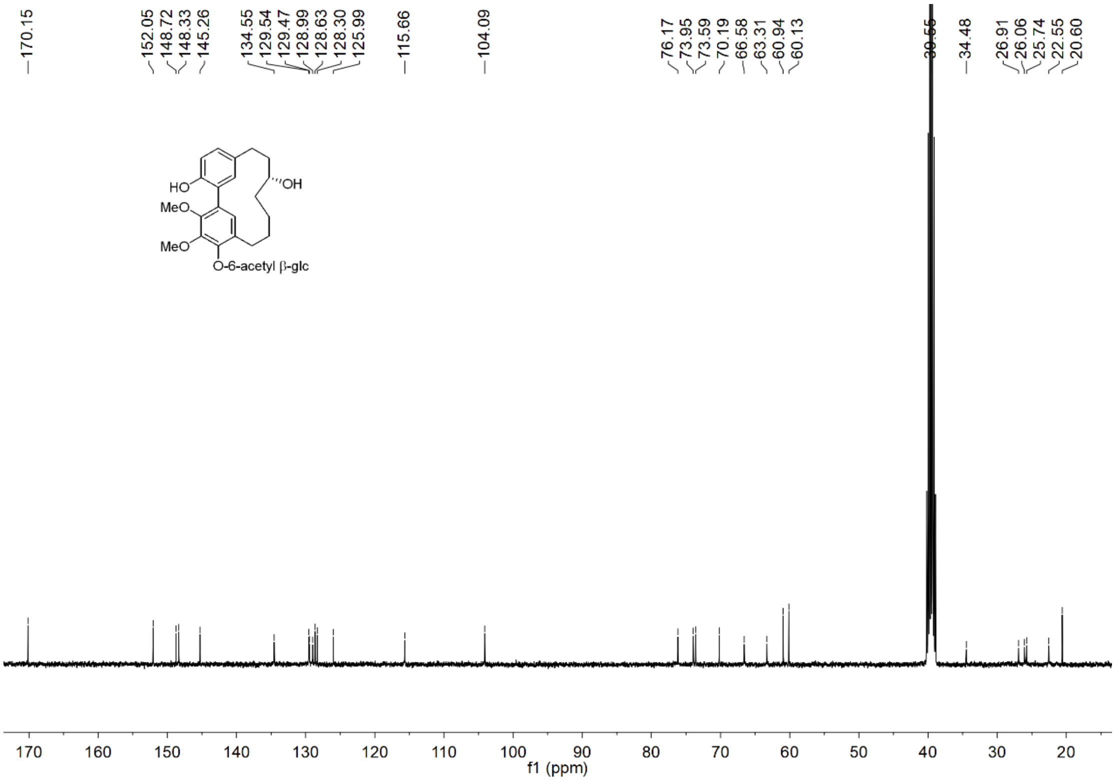

Figure S22 ^{13}C NMR spectra of **8** in CD_3OD

((2*S*,3*S*,4*S*,6*S*)-6-(((*R*)-2^6,5-dihydroxy-1^5,1^6-dimethoxy-1,2(1,3)-dibenzenacyclononaphane-14-yl)oxy)-3,4,5-trihydroxytetrahydro-2H-pyran-2-yl)methyl acetate, 8). ^{1}H NMR (400 MHz, CD_3OD): δ 8.98 (1H, s, OH), 6.98 (1H, dd, *J* = 8.2, 2.4 Hz, H-15), 6.94 (1H, d, *J* = 2.4 Hz, H-18), 6.76 (1H, d, *J* = 8.2 Hz, H-16), 6.61 (1H, s, H-19), 4.81 (1H, d, *J* = 7, H-1’), 4.21 (2H, m, H-6’), 3.85 (3H, s, 4-OMe), 3.82 (3H, s, 3-OMe), 3.60 (1H, m, H-11), 3.12-3.28 (4H, m, H-2’, 3’, 4’, 5’), 2.75 (2H, m, H-13), 2.55 (2H, m, H-7), 2.16 (1H, m, H-12a), 1.89 (3H, s, H-8’), 1.76 (2H, m, H-8), 1.66 (1H, m, H-10a), 1.52 (1H, m, H-12b), 1.29-1.38 (3H, m, H-9, H-10b); ^{13}C NMR (100 MHz, CD_3OD): δ 170.2 (7’), 152.1 (C-17), 148.7 (C-5), 148.3 (C-3), 145.3 (C-4), 134.6 (C-18), 129.5 (C-14), 129.5 (C-15), 129.0 (C-19), 128.6 (C-1), 128.3 (C-2), 126.0 (C-6), 115.7 (C-16), 104.1 (C-1’), 76.2 (C-5’), 74.0 (C-3’), 73.6 (C-2’), 70.2 (C-4’), 66.6 (C-11), 63.3 (C-6’), 60.9 (4-OMe), 60.1 (3-OMe), 39.6 (C-10), 34.5 (C-12), 26.9 (C-13), 26.1 (C-7), 25.7 (C-8), 22.6 (C-9), 20.6 (C-8’). $[\alpha]_D^{25}$ = -22.8 (*c* = 0.1, MeOH), ESI-MS: *m/z* 563.2 $[M + H]^+$.

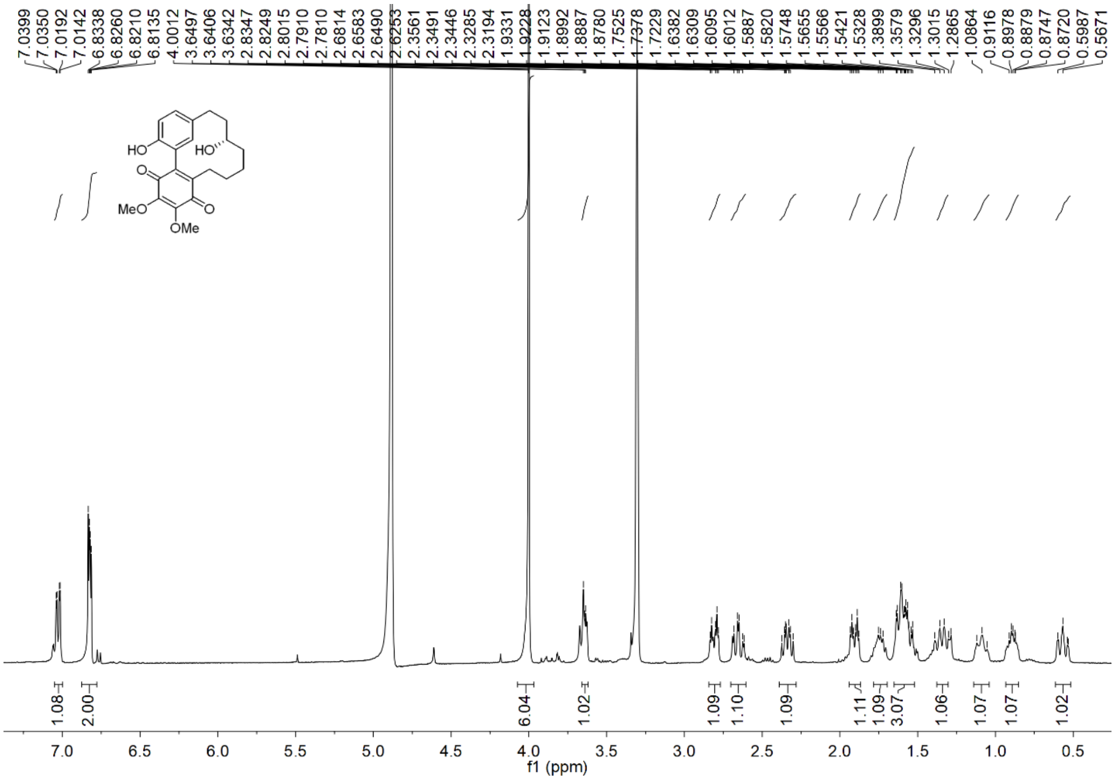

Figure S23. ^{1}H NMR spectra of **9** in CD_3OD

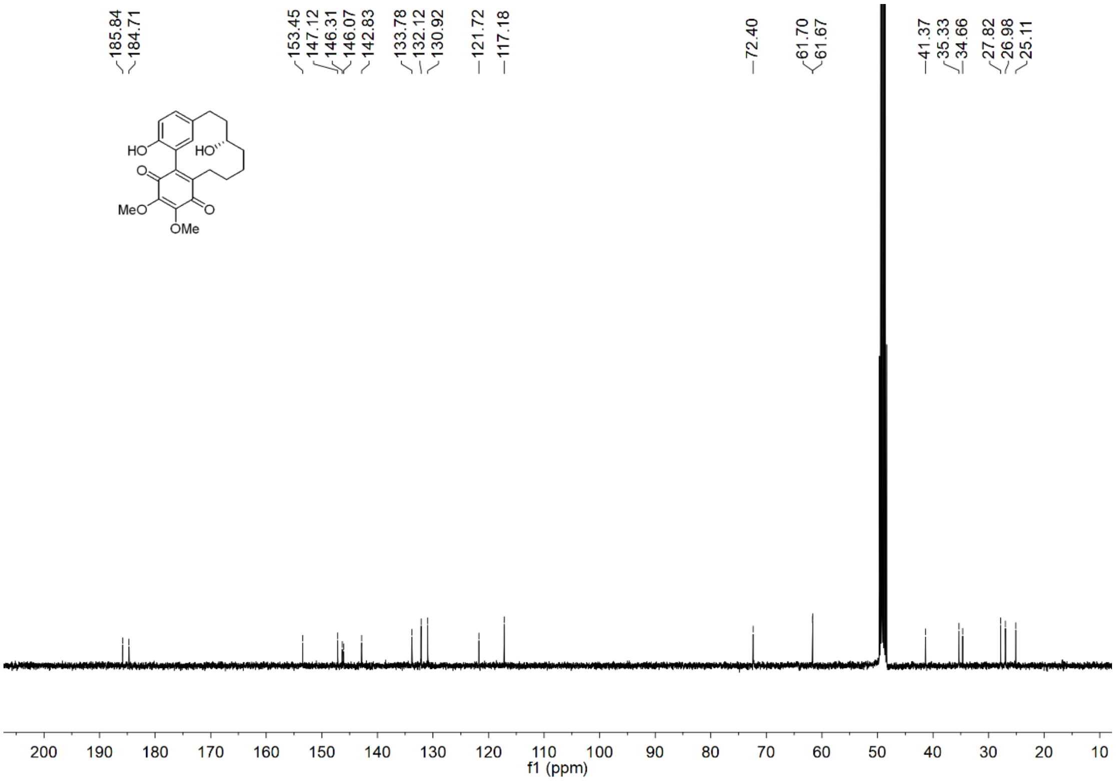

Figure S24. ^{13}C NMR spectra of **9** in CD_3OD

(*R*)-1^6,7-dihydroxy-2^4,2^5-dimethoxy-1(1,3)-benzena-2(1,2)-cyclohexanacyclononaphane-2^1,2^4-diene-2^3,2^6-dione (Actinidione, 9) (Guo et al. 2006). ^{1}H NMR (400 MHz, CD_3OD): δ 7.04 (1H, dd, *J* = 8.2, 2.2 Hz, H-16), 6.83 (1H, d, *J* = 8.2 Hz, H-17), 6.82 (1H, d, *J* = 1.6 Hz, H-19), 4.01, 4.00 (each 3H, s, 4 and 5-OMe), 3.65 (1H, m, H-12), 2.83 (1H, dt, *J* = 13.9, 3.6 Hz, H-14a), 2.68 (1H, td, *J* = 13.4, 3.2 Hz, H-14b), 2.37 (1H, m, H-9a), 1.94 (1H, dt, *J* = 13.3, 4.0 Hz, H-13a), 1.78 (1H, m, H-9a), 1.53-1.64 (3H, m, H-10a, H-8b, H-13b), 1.38 (1H, m, H-9b), 1.12 (1H, m, H-10b), 0.93 (1H, m, H-11a), 0.89 (1H, t, *J* = 12.7 Hz, H-11b); ^{13}C NMR (100 MHz, CD_3OD): δ 185.8 (C-6), 184.7 (C-3), 153.5 (C-18), 147.1 (C-7), 146.3 (C-4), 146.1 (C-5), 142.8 (C-2), 133. 8(C-15), 132.1 (C-19), 130.9 (C-16), 121.7 (C-1), 117.2 (C-17), 72.4 (C-12), 61.7 (5-OMe), 61.7 (4-OMe), 41.4 (C-13), 35.3 (C-11), 34.7 (C-14), 27.8 (C-9), 27.0 (C-8), 25.1(C-10). $[\alpha]_D^{25}$ = -19.2 (*c* = 0.1, MeOH), ESI-MS: *m/z* 373.2 $[M + H]^+$.

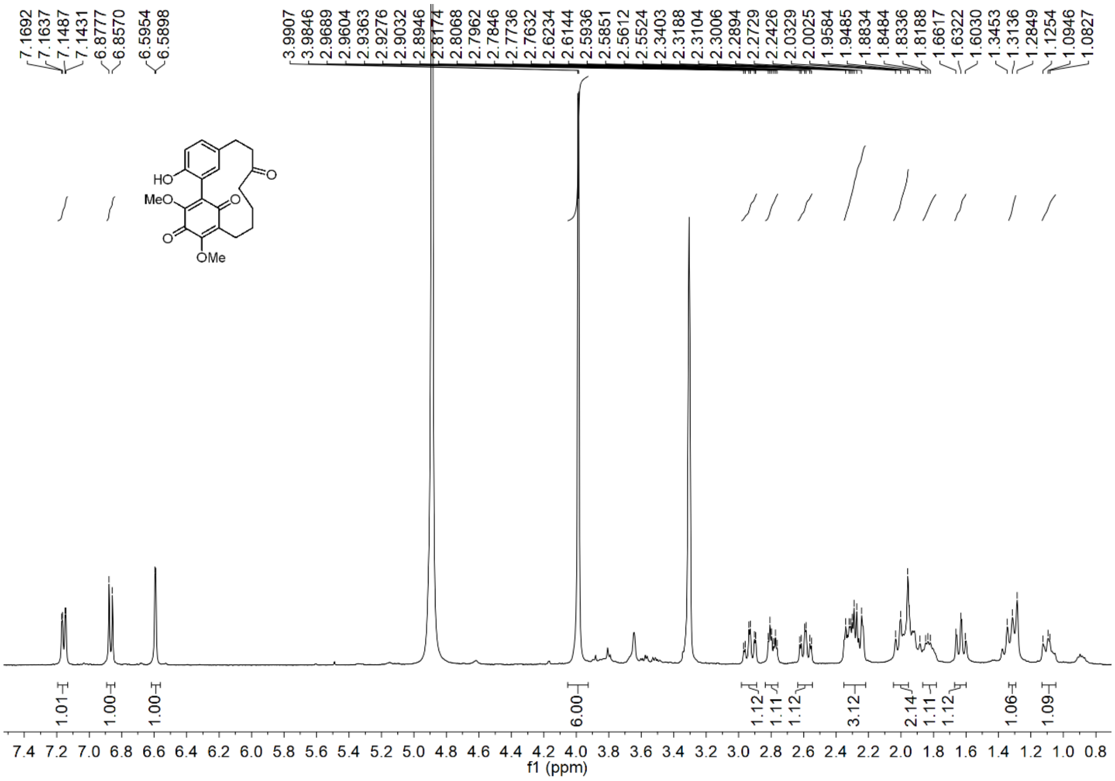

Figure S25. ^{1}H NMR spectra of **10** in CD_3OD

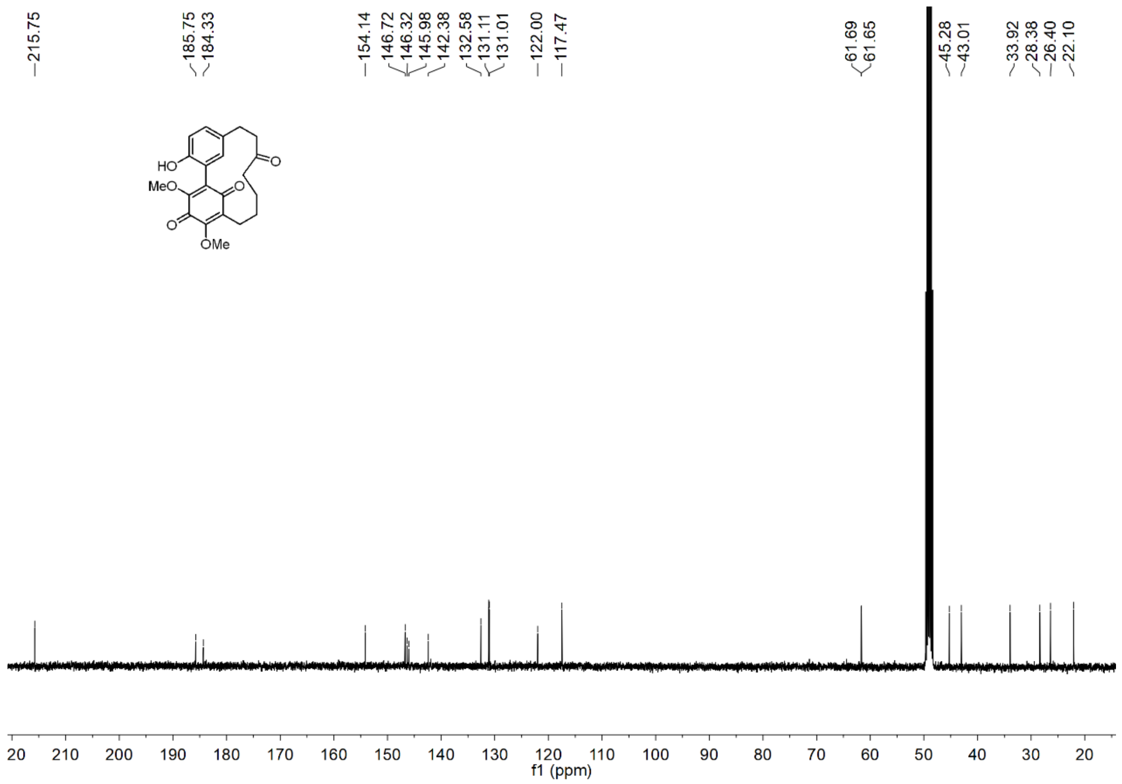

Figure S26. ^{13}C NMR spectra of **10** in CD_3OD

16-hydroxy-2^4,2^6-dimethoxy-1(1,3)-benzena-2(1,3)-cyclohexanacyclononaphane-2^1(2^6),2^3-diene-2^2,2^5,7-trione (**Rubanone**,**10**)(Wang, Liu, and Feng 2008). ^{1}H NMR (400 MHz, CD_3OD): δ 7.17(1H, dd, *J* = 2.2, 8.2 Hz, H-15), 6.88 (1H, d, *J* = 8.2 Hz, H-16), 6.60(1H, d, *J* = 2.2 Hz, H-18), 3.99, 3.98(each 3H, s, 3 and 4-OMe), 2.97 (1H, dt, *J* = 3.5, 13.1 Hz, H-13a), 2.81 (1H, dt, *J* = 4.2, 13.3 Hz, H-13b), 2.62 (1H, dt, *J* = 3.6, 11.9 Hz, H-12a), 2.24-2.34 (3H, m, H-7a, H-12b, H-10a), 1.90-2.03 (2H, m, H-9a, H-10b), 1.87 (1H, m, H-8a), 1.66 (1H, t, *J* = 11.8 Hz, H-7b), 1.32 (1H, m, H-8b), 1.13 (1H, m, H-9b); ^{13}C NMR (100 MHz, CD_3OD): δ 215.8 (C-11), 185.8 (C-19), 184.3 (C-4), 154.1 (C-17), 146.7 (C-6), 146.3 (C-3), 146.0 (C-5), 142.4 (C-2), 132.6 (C-14), 131.1 (C-15), 131.0 (C-18), 122.0 (C-1), 117.5 (C-16), 61.7 (3-OMe), 61.7 (5-OMe), 45.3 (C-12), 43.0 (C-10), 33.9 (C-13), 28.4 (C-8), 26.4 (C-7), 22.1 (C-9). ESI-MS: *m/z* 371.1 $[M + H]^+$.

Begley, M. J., R. V. Campbell, L. Crombie, B. Tuck, and D. A. Whiting. 1971. "Constitution and absolute configuration of meta,meta-bridged, strained biphenyls from *Myrica nagi*; X-ray analysis of 16-Bromomyricanol." *Journal of the Chemical Society C-Organic* (21):3634. doi: DOI 10.1039/j39710003634.

Curtiss, R., 3rd, S. Y. Wanda, B. M. Gunn, X. Zhang, S. A. Tinge, V. Ananthnarayan, H. Mo, S. Wang, and W. Kong. 2009. "*Salmonella enterica* serovar typhimurium strains with regulated delayed

attenuation in *vivo*." *Infect Immun* 77 (3):1071-1082. doi: 10.1128/IAI.00693-08.

Guo, H., B. G. Li, Z. J. Wu, and G. L. Zhang. 2006. "Lupane triterpenoids and a diarylheptanoid from *Clematoclethra actinidioides*." *Planta Med* 72 (2):180-183. doi: 10.1055/s-2005-873187.

Inoue, T., Y. Arai, and M. Nagai. 1984. "Diarylheptanoids in the bark of *Myrica rubra Sieb*. et Zucc." *Yakugaku Zasshi* 104 (1):37-41. doi: 10.1248/yakushi1947.104.1_37.

Li, J., C. Lv, W. Sun, Z. Li, X. Han, Y. Li, and Y. Shen. 2013. "Cytosporone B, an inhibitor of the type III secretion system of *Salmonella enterica* serovar Typhimurium." *Antimicrob Agents Chemother* 57 (5):2191-2198. doi: 10.1128/AAC.02421-12.

Martin, M. D., L. Calcul, C. Smith, U. K. Jinwal, S. N. Fontaine, A. Darling, K. Seeley, L. Wojtas, M. Narayan, J. E. Gestwicki, G. R. Smith, A. B. Reitz, B. J. Baker, and C. A. Dickey. 2015. "Synthesis, stereochemical analysis, and derivatization of myricanol provide new probes that promote autophagic tau clearance." *ACS Chem Biol* 10 (4):1099-109. doi: 10.1021/cb501013w.

Nagai, Masahiro, Junko Dohi, Motohiko Morihara, and Nobuko Sakurai. 1995. "Diarylheptanoids from *Myrica gale var. tomentosa* and revised structure of Porson." *CHEMICAL & PHARMACEUTICAL BULLETIN* 43 (10):1674-1677. doi: 10.1248/cpb.43.1674.

Sun, Dawang, Zuchun Zhao, Herbert Wong, and Yeap Foo Lai. 1988. "Tannins and other phenolics from Myrica esculenta bark." *Phytochemistry* 27 (2):579-583.

Tene, M., H. K. Wabo, P. Kamnaing, A. Tsopmo, P. Tane, J. F. Ayafor, and O. Sterner. 2000. "Diarylheptanoids from *Myrica arborea*." *Phytochemistry* 54 (8):975-978. doi: 10.1016/s0031-9422(00)00164-3.

Wang, D. Y., E. G. Liu, and Y. J. Feng. 2008. "A new diarylheptanoid from the bark of *Myrica rubra*." *Chinese Chem Lett* 19 (5):547-549. doi: 10.1016/j.cclet.2008.03.004.

Whiting, D. A., and A. F. Wood. 1980. "Total syntheses of the meta,meta-bridged biphenyls (±)-myricanol and myricanone, and of an isomeric biphenyl ether, a 14-oxa[7,1]metaparacyclophane." *Cheminform* 11 (19).
